# Hard ticks (Acari: Ixodidae) associated with birds in Europe: Review of literature data

**DOI:** 10.3389/fvets.2022.928756

**Published:** 2022-08-25

**Authors:** Gergő Keve, Attila D. Sándor, Sándor Hornok

**Affiliations:** ^1^Department of Parasitology and Zoology, University of Veterinary Medicine, Budapest, Hungary; ^2^ELKH-ÁTE Climate Change: New Blood-Sucking Parasites and Vector-Borne Pathogens Research Group, Budapest, Hungary; ^3^Department of Parasitology and Parasitic Diseases, University of Agricultural Sciences and Veterinary Medicine, Cluj-Napoca, Romania

**Keywords:** aves, biogeography, ectoparasite, host-parasite relationships, vector

## Abstract

Hard ticks (Acari: Ixodidae) are considered the most important transmitters of pathogens in the temperate zone that covers most of Europe. In the era of climate change tick-borne diseases are predicted to undergo geographical range expansion toward the north through regions that are connected to southern areas of the continent by bird migration. This alone would justify the importance of synthesized knowledge on the association of tick species with avian hosts, yet birds also represent the most taxonomically and ecologically diverse part of urban vertebrate fauna. Birds frequently occur in gardens and near animal keeping facilities, thus playing a significant role in the dispersal of ticks and tick-borne pathogens in synanthropic environments. The primary aim of this review is to provide a comprehensive reference source (baseline data) for future studies, particularly in the context of discovering new tick-host associations after comparison with already published data. The records on the ixodid tick infestations of birds were assessed from nearly 200 papers published since 1952. In this period, 37 hard tick species were reported from 16 orders of avian hosts in Europe. Here we compile a list of these tick species, followed by the English and Latin name of all reported infested bird species, as well as the tick developmental stage and country of origin whenever this information was available. These data allowed a first-hand analysis of general trends regarding how and at which developmental stage of ticks tend to infest avian hosts. Five tick species that were frequently reported from birds and show a broad geographical distribution in the Western Palearctic (*Ixodes arboricola, I. frontalis, I. ricinus, Haemaphysalis concinna* and *Hyalomma marginatum*) were also selected for statistical comparisons. Differences were demonstrated between these tick species regarding their association with bird species that typically feed from the ground and those that rarely occur at the soil level. The ecology of these five bird-infesting tick species is also illustrated here according to avian orders, taking into account the ecology (habitat type) and activity (circadian rhythm and feeding level) of most bird species that represent a certain order.

## Background

Hard ticks (Acari: Ixodidae) are considered the most important vectors (transmitters) of pathogens under the temperate zone (1) including most of Europe. In the era of climate change (global warming), vector-borne diseases in general, and tick-borne disease in particular are predicted to undergo dramatic changes in geographical distribution (2, 3) mostly implying range expansion of tick species toward more northern latitudes. In such scenarios birds may play the most important role in tick dispersal over both short and long distances due to their migratory habits (4). Passeriform birds can carry significant numbers of ticks [in a large scale study this was up to 20 ticks per bird: (5)]. These ticks are mostly larvae and nymphs of generalist (non-ornithophilic) tick species. These ticks may remain on their avian host for a period raging from a few days (in case of larvae of three-host tick species exemplified by *Ixodes ricinus*) to even 5 weeks or longer [in case of two-host ticks species, e.g., *Hyalomma rufipes*: (6)]. While most studies focus on tick species carried by birds during their spring migration from the southern wintering grounds toward the north in Europe, tick “transportation” by birds may equally be important in western, eastern, and north-to-southern directions (5).

If thermophilic ticks carried by birds from south to north detach after engorgement, and find themselves in a suitable (warm enough) environment, they can molt to the next developmental stage. These ticks may then survive through mild winters, potentially establishing new, northern populations (7). This may contribute to the increased risks and evident changes of tick-borne pathogen transmission due to climate warming and tick dispersal by migratory birds, even in northern Europe (8).

On the other hand, several bird-specialist (ornithophilic) *Ixodes* species occur in the Western Palearctic, most of which associate with sea birds in Western Europe (e.g., *I. uriae, I. caledonicus, I. rothschildi*). These ticks occur in and near avian nests, or are frequently endophilic and live in tree holes or burrows inhabited by nesting birds, particularly in the subgenus *Pholeoixodes* (e.g., *I. arboricola, I. lividus*) (9). Since the avian hosts of tick species are expected to be less affected by climate change in terms of their geographical distribution, ornithophilic ticks will likely undergo less significant changes in their geographical occurrence than generalist tick species carried by birds. In addition, ornithophilic ticks seldom infest humans or domestic mammals [e.g., *I. frontalis*: (10)] and thus may have a less important epidemiological role from a veterinary-medical point of view.

This review focuses on hard ticks reported from birds in Northern, Western, Central, and Southern Europe, in the hope that the checklist will provide useful baseline information for future scientific research in the topics of ticks and tick-borne pathogens. Nearly 700 scientific papers published since 1952 were checked for this review. Soft ticks (Acari: Argasidae) were not included in this review, because in the majority of cases these are reported from the environment and thus data on their association with avian host species are not representative.

## Methods

The primary corpus of publications used in this review was collated with database search using the following keywords: “ticks” OR “Ixodidae” OR “*Ixodes*” OR “*Hyalomma*” OR “*Haemaphysalis*” AND “birds” AND “Europe,” followed by a search with names of bird-specialist tick species (*I. arboricola, I. caledonicus, I. eldaricus, I. festai, I. frontalis, I. lividus, I. rothschildi, I. unicavatus* and *I. uriae*). The following databases were used: Web of Science, Zoological Record, and Google Scholar. These records were imported into an Excel file, followed by screening the publications and references cited within. After exclusion of duplicates, we extracted each individual bird host-tick record from these references, noting the location (country), host and parasite species and developmental stage of ticks.

Within Prostriata and Metastriata, tick species names are arranged alphabetically and are used sensu Guglielmone et al. (10). *Ixodes redikorzevi* is considered as a synonym of *I. acuminatus*. Data were only included in the checklist if the tick collected from a bird was reported as identified to the species level. E.g., in Norway a rare finding was mentioned, the occurrence of a *Dermacentor* sp. on a willow warbler (*Phylloscopus trochilus*) (11), but this was excluded from the main text.

Bird species as hosts of a certain tick species are listed alphabetically according to their international English name (https://bou.org.uk/british-list/bird-names/). When higher avian taxa are mentioned, these follow the phylogenetic order based on Kuhl et al. (12), starting with Passeriformes. Genus names are not abbreviated in the headings, owing to the high number and mixed usage of host and tick Latin names, also taking into account that scientists from a broad range of biology-related fields may use this checklist. Whenever a bird species was mentioned by its binominal name in an article, this was used for identification, even if the English name was also written in the text. English and Latin host species names are followed by tick developmental stages if this information was available (L: larva, N: nymph, M: male, F: female, A: adult [where there was no information about the sex of the adult tick)]. The abbreviation “NA” is used to indicate that no data were available about the sex and developmental stage of ticks collected from birds. If a tick species was reported from bird(s) in a country without mentioning avian host species, an exclamation mark (!) follows the country name in the list. If tick species infesting a certain bird species are mentioned in a reference but only some of the data inform about the tick developmental stage, only these were incorporated into the text. It is also noteworthy that in some reports blood meal analysis allowed the identification of previous tick hosts. In addition, data on ticks reported from bird nests are included and marked in the text as “in nest”: these ticks probably also originate from or can associate with birds.

The geographical area covered by this review is in the Western Palearctic, excluding North Africa, the Middle East, Belarus and Russia but including Ukraine. Cyprus however, is also considered. Not just because it is partially European territory, but due to the fact that the island has a high epidemiological significance concerning the aim of this manuscript. Regarding geographical names, old references often refer to Czechoslovakia, which no longer exists. In cases when it was unambiguous whether the samples came from the Czech Republic or Slovakia, the country is mentioned as such. To maintain the user friendliness of this checklist the broader Palearctic distribution, general ecology, and vector role of tick species are not mentioned. This is because this information is available in other sources [e.g., (9)].

## Review of literature data

### Prostriata

#### Ixodes acuminatus

Overview: *Ixodes acuminatus* is distributed in temperate and Mediterranean Europe (8). Accordingly, this tick species is occasionally found on birds in South-European countries. Based on literature data it is mainly a parasite of passeriform birds (from this order 15 species have been shown as hosts) but has also been found twice on galliform birds.

Passeriformes: 15; Galliformes: 2

Hosts:

- Bearded reedling – *Panurus biarmicus* [N: (13)]
- Common blackbird – *Turdus merula* [F: (14–16); NA: (17, 18)]
- Common chaffinch – *Fringilla coelebs* [L: (14); N: (14)]
- Common pheasant – *Phasianus colchicus* [A: (19)]
- Common redstart – *Phoenicurus phoenicurus* [L: (14)]
- Eurasian blue tit- *Cyanistes caeruleus* [N: (14)]
- Eurasian magpie – *Pica pica* [L: (14); N: (14)]
- European goldfinch – *Carduelis carduelis* [F: (14)]
- European robin – *Erithacus rubecula* [N: (13)]
- Fieldfare -*Turdus pilaris* [NA: (14)]
- Garden warbler – *Sylvia borin* [N: (20)]
- Great tit – *Parus major* [L: (14); N: (13, 14)]
- House sparrow – *Passer domesticus* [NA: (14)]
- Red-legged partridge – *Alectoris rufa* [A: (19)]
- Redwing – *Turdus iliacus* [N: (21)]
- Song thrush – *Turdus philomelos* [F: (16)]
- Winter wren – *Troglodytes troglodytes* [L: (21); N: (21)]

Distribution of reported cases: Greece (20), Italy (19, 22!), Romania (13, 14, 23!), Cyprus (16), Portugal (15, 17, 21), France (18).

#### Ixodes arboricola

Overview: *Ixodes arboricola* is widespread throughout Europe. As its English name suggests, the Tree-hole tick is primarily a parasite of hole-nesting birds (8). Literature data usually support this theory, as *I. arboricola* was mostly found on passeriform (35 species), strigiform (5 species), piciform (1 species), and columbiform (1 species) birds. The fact that other, non-hole-nesting predators (2 falconiform and 1 accipitriform species) have been described as hosts, does not contradict the former statement, as these species can become infected with ticks by getting in contact with their prey.

Passeriformes: 34; Strigiformes: 5; Falconiformes: 2; Columbiformes: 1; Piciformes: 1; Accipitriformes: 1

Hosts:

- Barn owl – *Tyto alba* [L: (24); NA: (25, 26)]
- Barn swallow –* Hirundo rustica* [NA: (26)]
- Boreal owl – *Aegolius funereus* [N: (27 in nest)]
- Coal tit – *Periparus ater* [N: (28); NA: (26, 29, 30)]
- Collared flycatcher – *Ficedula albicollis* [N: (31, 32); M: (31, 32 in nest); F: (32^a^); NA: (33)]
- Common blackbird – *Turdus merula* [L: (15); N: (13, 14, 34); NA: (30, 35)]
- Common kestrel- *Falco tinnunculus* [F: (34)]
- Common redstart – *Phoenicurus phoenicurus* [L: (14, 31); N: (14); NA: (33)]
- Common starling – *Sturnus vulgaris* [L: (31, 34, 36, 37); N: (31, 34, 36, 37); M: (37); F: (31, 34, 36, 37); NA: (14, 26, 30, 33, 38^a^ in nest)]
- Common wood pigeon – *Columba palumbus* [L: (34)]
- Eurasian blue tit- *Cyanistes caeruleus* [L: (14, 28, 39–42); N: (13, 15, 28, 31, 40–45); M: (32 in nest); F: (31, 32, 40, 42, 43); NA: (17, 25, 29, 33, 38, 46)]
- Eurasian bullfinch – *Pyrrhula pyrrhula* [NA: (30)]
- Eurasian jay – *Garrulus glandarius* [A: (47)]
- Eurasian nuthatch – *Sitta europaea* [L: (21, 46); N: (21, 28, 45–47); A: (46); M: (32 in nest); NA: (26, 29, 33, 48)]
- Eurasian penduline tit – *Remiz pendulinus* [L: (49 in nest)]
- Eurasian pygmy owl – *Glaucidium passerinum* [L: (31)]
- Eurasian reed warbler – *Acrocephalus scirpaceus* [NA: (30)]
- Eurasian siskin – *Carduelis spinus* [N: (41)]
- Eurasian tree sparrow – *Passer montanus* [NA: (26, 30, 38)]
- Eurasian treecreeper – *Certhia familiaris* [L: (33), NA: (29)]
- European greenfinch – *Carduelis chloris* [L: (42)]
- European pied flycatcher – *Ficedula hypoleuca* [N: (31, 45); NA: (30, 38)]
- European robin – *Erithacus rubecula* [L: (13, 14); N: (11, 13, 14, 28, 31); NA: (30)]
- European serin – *Serinus serinus* [N: (21); F: (21)]
- Great spotted woodpecker – *Dendrocopos major* [NA: (47)]
- Great tit – *Parus major* [L: (14, 28, 31, 34, 36, 40–42, 44, 50, 51); N: (13– 15, 17, 21, 24, 28, 31, 32, 34, 36, 40–43, 45, 50, 51); M: (32^a^ in nest); F: (24, 32^a^, 40, 41, 43, 52, 53); NA: (17, 25, 29, 33, 38, 46)]
- House sparrow – *Passer domesticus* [NA: (26, 30)]
- Little owl – *Athene noctua* [N: (34); F: (34); NA: (30)]
- Long-tailed tit – *Aegithalos caudatus* [L: (21)]
- Marsh tit – *Poecile palustris* [L: (28, 42); N: (42); NA: (29, 30)]
- Northern goshawk – *Accipiter gentilis* [L: (34)]
- Peregrine falcon – *Falco peregrinus* [L: (54); N: (54); F: (54)]
- Rook – *Corvus frugilegus* [L: (55)]
- Sand martin – *Riparia riparia* [N: (24 in nest); F: (24 in nest); NA: (30)]
- Short-toed treecreeper – *Certhia brachydactyla* [L: (15); NA: (17)]
- Song thrush – *Turdus philomelos* [L: (13); N: (11, 13)]
- Spotless starling – *Sturnus unicolor* [L: (17); NA: (17)]
- Spotted flycatcher – *Muscicapa striata* [N: (14); F: (31)]
- Tawny owl -*Strix aluco* [L: (31); NA: (26)]
- Western jackdaw – *Corvus monedula* [L: (55); N: (31); F: (31); NA: (30, 48)]
- Willow tit – *Poecile montana* [N: (41); NA: (30)]
- Willow warbler – *Phylloscopus trochilus* [NA: (30)]
- Winter wren – *Troglodytes troglodytes* [N: (41); NA: (30)]
- Yellowhammer – *Emberiza citrinella* [NA: (25)]

Distribution of reported cases: Sweden (31, 36, 56!), United Kingdom (24, 25, 30, 46, 53), Czech Republic (28, 29, 32, 57!), Slovakia (26, 32^a^, 33, 49), Netherlands (43), Denmark (37), Norway (11), Belgium (40, 45, 48, 50), Romania (13, 14, 23!, 55), Ukraine (52), Portugal (15, 17, 21), Poland (26, 41, 51), Spain (42, 44), Switzerland (34), Germany (38, 54), Hungary (47), Croatia (35), Belarus (38^a^).

#### Ixodes berlesei

Overview: According to literature data, *I. berlesei* was reported from birds in France. Unfortunately, the source article (18) is not accurate about the host species, but we do know that they belong to the Columbiformes order.

Columbiformes:1

Distribution of reported cases: France (18!).

#### Ixodes caledonicus

Overview: *Ixodes caledonicus* is an ornithophilic tick species (10). It is primarily a parasite of the northern European bird fauna. Despite the limited data available, the host range appears to be broad: it has been reported from 6 passeriform, 3 falconiform, 1 caprimulgiform, and one procellariiform bird species.

Passeriformes: 6; Falconiformes: 3; Caprimulgiformes: 1; Procellariiformes: 1; Columbiformes: 1

Hosts:

- Common kestrel- *Falco tinnunculus* [NA: (30)]
- Common redstart – *Phoenicurus phoenicurus* [N: (24)]
- Common starling – *Sturnus vulgaris* [NA: (30)]
- Common swift – *Apus apus* [NA: (26)]
- Gyrfalcon – *Falco rusticolus* [L: (58); N: (58); A: (58); NA: (30)]
- Hooded crow – *Corvus cornix* [NA: (30)]
- Northern fulmar – *Fulmarus glacialis* [F: (59); NA: (30)]
- Northern raven – *Corvus corax* [NA: (30)]
- Peregrine falcon – *Falco peregrinus* [N: (31)]
- Red crossbill – *Loxia curvirostra* [NA: (30)]
- Rock dove –* Columba livia* [NA: (59^a^)]
- Western jackdaw – *Corvus monedula* [NA: (30)]

Distribution of reported cases: United Kingdom (24, 30), Faroe Islands (59), Sweden (31), Poland (26), Norway (30!), Germany (30!), Iceland (58), NA (59^a^).

#### Ixodes canisuga

Overview: *Ixodes canisuga* is primarily a parasite of mammals (8) and it is relatively rare on birds. So far, it has been found on 5 passeriform and on 2 strigiform hosts.

Passeriformes: 5; Strigiformes: 2

Hosts:

- Common starling – *Sturnus vulgaris* [NA: (30)]
- Eurasian blue tit- *Cyanistes caeruleus* [NA: (30)]
- Eurasian eagle-owl – *Bubo bubo* [N: (60)]
- Eurasian tree sparrow – *Passer montanus* [NA: (30)]
- Great tit – *Parus major* [NA: (30)]
- Little owl – *Athene noctua* [L: (24); N: (24); F: (24)]
- Sand martin – *Riparia riparia* [M: (24 in nest); F: (24 in nest)]

Distribution of reported cases: United Kingdom (24, 30), Germany (61!), Portugal (60).

#### Ixodes eldaricus

Overview: *Ixodes eldaricus* is a rare, poorly known tick species. The only available data about this parasite feeding on European birds are from Poland and Cyprus, where this tick species was reported from four passeriform bird species in total

Passeriformes: 4

Hosts:

- Common blackbird – *Turdus merula* [N: (16)]
- Dunnock – *Prunella modularis* [F: (62)]
- European robin – *Erithacus rubecula* [M: (62); F: (62)]
- Tree pipit – *Anthus trivialis* [N: (16)]

Distribution of reported cases: Poland (62), Cyprus (16).

#### Ixodes festai

Overview: *Ixodes festai* is a poorly known species that was reported from 5 passeriform birds, mostly in Central European countries.

Passeriformes: 5

Hosts:

- Common blackbird – *Turdus merula* [N: (34); F: (34, 51, 63); NA: (64)]
- Common chaffinch – *Fringilla coelebs* [F: (34)]
- Dunnock – *Prunella modularis* [N: (34); F: (65)]
- European greenfinch – *Carduelis chloris* [F: (65)]
- Song thrush – *Turdus philomelos* [M: (64); F: (63, 64)]

Distribution of reported cases: Italy (63, 64), Hungary (65), Switzerland (34), Poland (51).

#### Ixodes frontalis

Overview: *Ixodes frontalis* has a pan-European distribution. However, it appears to be more frequent in warmer regions. It is an ornithophilic tick species that rarely feeds on other, exceptional hosts (10). As such, it is commonly found on birds, mostly on Passeriformes, but the host range appears to be broad.

Passeriformes: 56; Charadriiformes: 1; Accipitriformes: 4; Galliformes: 4; Falconiformes: 1; Coraciiformes: 1; Gruiformes: 3; Columbiformes: 2; Strigiformes: 2

Hosts:

- Barn owl – *Tyto alba* [F: (25)]
- Black redstart – *Phoenicurus ochruros* [L: (66); NA: (35)]
- Black-headed gull – *Chroicocephalus ridibundus* [F: (67)]
- Bohemian waxwing – *Bombycilla garrulus* [NA: (35)]
- Booted eagle – *Hieraaetus pennatus* [A: (60)]
- Carrion crow – *Corvus corone* [NA: (68)]
- Cetti's warbler – *Cettia cetti* [L: (21)]
- Chicken – *Gallus gallus domesticus* [NA: (46)]
- Coal tit – *Periparus ater* [NA: (30, 68)]
- Common blackbird – *Turdus merula* [L: (15, 17, 20, 21, 34, 39, 47, 66, 67, 69–74); N: (15–17, 21, 24, 39, 43, 47, 60, 66–76); M: (71, 75); F: (21, 25, 44, 66, 67, 69–74, 77); A: (78); NA: (48, 68)]
- Common buzzard – *Buteo buteo* [N: (60); A: (60)]
- Common chaffinch – *Fringilla coelebs* [L: (21, 24, 66, 69); N: (15, 21, 69); F: (20, 21, 66, 70, 79), (79); NA: (30, 68)]
- Common chiffchaff – *Phylloscopus collybita* [L: (16, 21, 66, 71); N: (21, 80)]
- Common firecrest – *Regulus ignicapilla* [N: (15, 21)]
- Common kestrel – *Falco tinnunculus* [F: (74); NA: (46, 68)]
- Common kingfisher – *Alcedo atthis* [F: (67)]
- Common linnet – *Carduelis cannabina* [NA: (30)]
- Common moorhen – *Gallinula chloropus* [F: (81)]
- Common nightingale – *Luscinia megarhynchos* [N: (71, 82); F: (21)]
- Common pheasant – *Phasianus colchicus* [NA: (68)]
- Common redstart – *Phoenicurus phoenicurus* [N: (67, 80)]
- Common reed bunting – *Emberiza schoeniclus* [L: (66)]
- Common starling – *Sturnus vulgaris* [NA: (48, 68)]
- Common whitethroat – *Sylvia communis* [N: (25, 53, 71, 75, 76, 80); F: (25, 53)]
- Common wood pigeon – *Columba palumbus* [F: (25); NA: (68)]
- Corn crake – *Crex crex* [NA: (68)]
- Dunnock – *Prunella modularis* [L: (66); F: (25, 53, 66)]
- Eurasian blackcap – *Sylvia atricapilla* [L: (16, 21, 66, 69, 71); N: (11, 21, 60, 65, 71, 80); F: (25, 53, 71); A: (47); NA: (48)]
- Eurasian blue tit – *Cyanistes caeruleus* [L: (15, 21, 25, 40, 53, 71); N: (40); F: (21, 40, 70, 71, 77); NA: (68)]
- Eurasian bullfinch – *Pyrrhula pyrrhula* [F: (25, 53)]
- Eurasian collared dove – *Streptopelia decaocto* [F: (25, 67, 81, 83)]
- Eurasian jay – *Garrulus glandarius* [L: (47); F: (21); A: (47); NA: (30, 68)]
- Eurasian magpie – *Pica pica* [NA: (68)]
- Eurasian nuthatch – *Sitta europaea* [NA: (68)]
- Eurasian reed warbler – *Acrocephalus scirpaceus* [L: (21, 67); N: (21, 70, 80); F: (21)]
- Eurasian sparrowhawk – *Accipiter nisus* [NA: (68)]
- Eurasian stonechat – *Saxicola torquatus* [NA: (68)]
- Eurasian tree sparrow – *Passer montanus* [F: (25); NA: (68)]
- Eurasian treecreeper – *Certhia familiaris* [NA: (30)]
- European goldfinch – *Carduelis carduelis*[(F: (74)]
- European greenfinch – *Carduelis chloris* [L: (15, 21, 67); F: (16, 21, 65, 67); NA: (68)]
- European pied flycatcher – *Ficedula hypoleuca* [NA: (30)]
- European robin – *Erithacus rubecula* [L: (15–17, 20, 21, 34, 39, 65– 67, 69, 71–73); N: (15, 17, 21, 34, 39, 41, 65, 66, 71–73, 84); M: (21); F: (16, 21, 25, 65, 73, 82)]
- Fieldfare -*Turdus pilaris* [F: (74); NA: (68)]
- Goldcrest – *Regulus regulus* [N: (41); NA: (30)]
- Great gray shrike – *Lanius excubitor* [NA: (68)]
- Great tit – *Parus major* [L: (15, 17, 21, 34, 40, 65, 66, 71); N: (15, 17, 21, 40); F: (21, 25, 40, 65); NA: (68)]
- Gray partridge – *Perdix perdix* [NA: (68)]
- Gray wagtail – *Motacilla cinerea* [N: (67)]
- Harris's hawk – *Parabuteo unicinctus* [F: (74)]
- House sparrow – *Passer domesticus* [L: (24); N: (25); F: (21, 24, 25, 67); NA: (68)]
- Lesser redpoll – *Carduelis cabaret* [F: (25, 70)]
- Long-eared owl – *Asio otus* [L: (67); N: (85); F: (85); NA: (68)]
- Long-tailed tit – *Aegithalos caudatus* [N: (21, 71); F: (25, 53, 71, 72)]
- Marsh tit – *Poecile palustris* [NA: (30)]
- Melodious warbler – *Hippolais polyglotta* [N: (21)]
- Mistle trush – *Turdus viscivorus* [NA: (48, 68)]
- Red-legged partridge – *Alectoris rufa* [M: (67); A: (86); NA: (68, 87)]
- Redwing – *Turdus iliacus* [L: (21, 70); N: (21, 67, 70, 73); A: (60); NA: (30, 68)]
- Ring ouzel – *Turdus torquatus* [F: (16); NA: (30)]
- Rook – *Corvus frugilegus* [NA: (30)]
- Sardinian warbler – *Sylvia melanocephala* [L: (15); N: (71); F: (67)]
- Scarlet-headed blackbird – *Amblyramphus holosericeus* [F: (25)]
- Sedge warbler – *Acrocephalus schoenobaenus* [F: (21)]
- Short-toed treecreeper – *Certhia brachydactyla* [L: (15, 21); N: (15, 21); F: (66)]
- Song thrush – *Turdus philomelos* [L: (15, 16, 21, 41, 65–67, 70–72, 80); N: (15, 16, 21, 34, 39, 41, 65, 66, 70–73, 80); F: (16, 21, 25, 44, 53, 70, 71, 77); NA: (68)]
- Subalpine warbler – *Sylvia cantillans* [N: (84)]
- Tree pipit – *Anthus trivialis* [NA: (30)]
- Water rail – *Rallus aquaticus* [F: (67); NA: (30)]
- Western yellow wagtail – *Motacilla flava* [L: (21); N: (16)]
- Whinchat – *Saxicola rubetra* [N: (84)]
- Willow tit – *Poecile montana* [NA: (30, 48)]
- Willow warbler – *Phylloscopus trochilus* [L: (16, 24, 73, 80); N: (80); F: (80)]
- Winter wren – *Troglodytes troglodytes* [L: (15, 17, 21, 66, 67, 73); N: (15, 17, 21, 31, 66); F: (25, 53); NA: (68)]

Distribution of reported cases: United Kingdom (24, 25, 30, 46, 53, 75–77, 80), France (67, 68, 81, 83), Spain (44, 66, 71, 72, 86, 87), Poland (39, 41), Greece (20), Germany (74), Netherlands (43, 70), Norway (11), Belgium (40, 48), Hungary (47, 65), Sweden (56!, 73), Portugal (Azores) (69), Italy (22!, 78, 84), Switzerland (79, 82), Moldova (88!), Portugal (15, 17, 21, 60, 85), Croatia (35), Cyprus (16).

#### Ixodes gibbosus

Overview: *Ixodes gibbosus* is a Mediterranean tick species, rarely reported from birds (8).

Passeriformes: 1

Hosts:

- Common blackbird – *Turdus merula* (L/N: (89)]

Distribution of reported cases: Greece (89).

#### Ixodes hexagonus

Overview: *Ixodes hexagonus* is a common parasite of European foxes and hedgehogs (8). In the United Kingdom, this tick was reported from 5 passeriform, 1 galliform, 1 falconiform, 1 columbiform and, 1 strigiform birds. Whereas, in Spain it was reported from 1 galliform bird. This tick species was also found on a bird in Germany but no information is available on the host species.

Passeriformes: 3; Galliformes: 2; Falconiformes: 1; Columbiformes: 1

Hosts:

- Chicken – *Gallus domesticus* [F: (25)]
- Common kestrel- *Falco tinnunculus* [NA: (30)]
- Common starling – *Sturnus vulgaris* [NA: (30)]
- Common wood pigeon – *Columba palumbus* [F: (46)]
- Eurasian blue tit- *Cyanistes caeruleus* [NA: (30)]
- Red-legged partridge – *Alectoris rufa* [NA: (90)]
- Winter wren – *Troglodytes troglodytes* [NA: (30)]

Distribution of reported cases: United Kingdom (25, 30, 46), Germany (61!), Spain (90).

#### Ixodes lividus

Overview: *Ixodes lividus* has a pan-European distribution, and it is the host-specific parasite of the sand martin (*Riparia riparia*). In Poland, it has been found on a Barn swallow (*Hirundo rustica*) as well.

Passeriformes: 2

Hosts:

- Barn swallow –* Hirundo rustica* [dNA: (26)]
- Sand martin – *Riparia riparia* [L: (31, 34, 53, 91 in nest, 92 in nest, 93–95 in nest, 96); N: (31, 34, 52, 53, 81, 91 in nest, 92 in nest, 93–95 in nest, 96); M: (31, 34, 91 in nest, 92 in nest, 95 in nest, 96 in nest); F: (31, 34, 48, 53, 91 in nest, 92 in nest, 95 in nest, 96 in nest, 97); A: (94, 96); NA: (25, 98–102)]

Distribution of reported cases: United Kingdom (25, 53, 97, 98), France (81), Portugal (99), Finland (96), Sweden (31, 103!) Germany (61!, 94), Lithuania (91, 92), Moldova (93), Ukraine (52), Czech Republic (95), Schwitzerland (34), Hungary (100, 101), Poland (26, 102), Belgium (48).

#### Ixodes persulcatus

Overview*: Ixodes persulcatus* is widely distributed in the Northern European region (Russia, Scandinavia, and the Baltic region) (8). According to literature data however, it should be considered as a rare parasite of birds in Europe. It was reported only from 2 passeriform bird species.

Passeriformes: 2

Hosts:

- Sedge warbler – *Acrocephalus schoenobaenus* [N: (104)]
- Willow warbler – *Phylloscopus trochilus* [N: (31)]

Distribution of reported cases: Estonia (104), Sweden (31).

#### Ixodes ricinus

Overview: *Ixodes ricinus* has a pan-European distribution, including countries of Southern, Western, Central, Eastern and Northern Europe. As outlined below, this tick species was reported from 99 passeriform bird species, and from species of further 12 avian orders. According to available data, the immature stages of this tick species appear to be the most frequent hard ticks feeding on European birds.

Passeriformes: 99; Galliformes: 8; Accipitriformes: 6; Ciconiiformes: 1; Anseriformes: 3; Gruiformes: 3; Cuculiformes: 1; Charadriiformes: 7; Falconiformes: 1; Columbiformes: 2; Strigiformes: 3; Piciformes: 4; Bucerotiformes: 1

Hosts:

- Barn swallow –* Hirundo rustica* [N: (31)]
- Barred warbler – *Sylvia nisoria* [L: (105); N: (36, 105, 106); NA: (47)]
- Bearded reedling – *Panurus biarmicus* [N: (13)]
- Black grouse – *Tetrao tetrix* [L: (107); N: (31, 107)]
- Black kite – *Milvus migrans* [NA: (47)]
- Black redstart – *Phoenicurus ochruros* [N: (14, 41, 73, 79)]
- Black stork – *Ciconia nigra* [N: (108)]
- Bluethroat – *Luscinia svecica* [L: (31, 34, 43, 109); N: (31, 70, 73, 105, 106, 110–113)]
- Blyth's reed warbler – *Acrocephalus dumetorum* [N: (104)]
- Bohemian waxwing – *Bombycilla garrulus* [NA: (14)
- Brambling – *Fringilla montifringilla* [L: (34, 73, 79, 114); N: (34, 36, 79, 114); NA: (115)]
- Canada goose – *Branta canadensis* [L: (107); N: (107)]
- Carrion crow – *Corvus corone* [N: (34); NA: (30)]
- Cetti's warbler – *Cettia cetti* [L: (15); N: (15); NA: (35)]
- Chicken – *Gallus gallus domesticus* [M: (34)]
- Coal tit – *Periparus ater* [L: (34, 116, 117); N: (117, 118); NA: (26, 29, 57, 115)]
- Collared flycatcher – *Ficedula albicollis* [N: (119); NA: (33)]
- Common blackbird – *Turdus merula* [L: (11, 13–15, 17, 21, 28, 31, 34, 36, 39, 41, 43, 44, 51, 57, 65, 66, 70, 73–76, 78, 79, 93, 105, 106, 109, 112–140); N: (11, 13–15, 17, 21, 24, 25, 28, 31, 34, 36, 39, 41, 43, 44, 51, 52, 57, 65, 66, 70–79, 93, 104–106, 108, 109, 111–142); M: (31); F: (13–15, 21, 52, 73, 76); A: (119); NA: (29, 33, 35, 46, 48, 143–145)]
- Common buzzard – *Buteo buteo* [N: (34); NA: (26, 33, 35, 46, 47)]
- Common chaffinch – *Fringilla coelebs* [L: (11, 14, 15, 17, 25, 34, 36, 39, 41, 43, 66, 70, 73, 76, 79, 93, 105, 109, 112–121, 124, 129, 133, 135– 140); N: (11, 14, 15, 34, 39, 41, 43, 52, 70, 71, 73, 78, 79, 93, 105, 109, 112^a^, 113, 114, 116, 117, 120, 121, 126, 129, 133, 135–140, 146); F: (17, 21, 79); NA: (33, 46–48, 57, 115, 123, 130, 144, 145, 147)]
- Common chiffchaff – *Phylloscopus collybita* [L: (34, 39, 41, 65, 71, 112, 113, 129, 139, 142); N: (13, 21, 28, 31, 39, 41, 65, 70, 73, 76, 80, 104, 109, 120, 128, 129, 133, 138, 140, 141); NA: (57, 143); NA: (59)]
- Common coot – *Fulica atra* [NA: (26)]
- Common crane – *Grus grus* [NA: (30)]
- Common cuckoo – *Cuculus canorus* [NA: (26)]
- Common firecrest – *Regulus ignicapilla* [N: (39); NA: (123)]
- Common grasshopper warbler – *Locustella naevia* [L: (122); N: (39, 104, 122); NA: (30)]
- Common house martin – *Delichon urbicum* [F: (34); NA: (30)]
- Common kestrel- *Falco tinnunculus* [L: (34); N: (34); NA: (14)]
- Common linnet – *Carduelis cannabina* [L: (76); N: (43, 70, 105, 106, 109, 112, 113); NA: (47)]
- Common nightingale – *Luscinia megarhynchos* [L: (34, 43, 65, 78, 128, 140, 148); N: (28, 34, 65, 71, 78, 128, 140, 148, 149); A: (140); NA: (14, 26, 33, 35)]
- Common pheasant – *Phasianus colchicus* [L: (24, 31, 150); N: (24, 31, 34, 43, 150); M: (34); NA: (14, 33, 46, 47)]
- Common quail – *Coturnix coturnix* [N: (75)]
- Common redpoll – *Carduelis flammea* [L: (105); N: (73); NA: (30)]
- Common redstart – *Phoenicurus phoenicurus* [L: (11, 31, 36, 39, 41, 43, 70, 76, 79, 105, 106, 119, 124, 130, 132, 133, 139); N: (11, 31, 36, 41, 43, 70, 73, 76, 79, 104–106, 110–113, 119, 124, 130, 132, 133, 141); NA: (33, 144)]
- Common reed bunting – *Emberiza schoeniclus* [L: (13, 65, 105); N: (28, 43, 70, 73, 104)]
- Common rosefinch – *Carpodacus erythrinus* [L: (28); N: (28, 104, 105, 109, 112, 113); NA: (26)]
- Common starling – *Sturnus vulgaris* [L: (14, 34, 36, 78, 105, 106, 109, 112, 113, 121, 133, 135, 137, 139); N: (14, 34, 36, 73, 78, 105, 106, 112, 113, 121, 133, 135, 137, 139, 146); A: (119); NA: (30, 33, 47, 48, 147)]
- Common whitethroat – *Sylvia communis* [L: (24, 31, 36, 43, 65, 70, 73, 75, 76, 105, 106, 109, 112, 113, 119, 122, 128, 132, 138); N: (28, 31, 34, 36, 41, 43, 53, 65, 70, 73, 75, 76, 79, 80, 104–106, 109, 110, 112^a^, 113, 122, 124, 128, 132); F: (31); NA: (33, 144)]
- Corn bunting – *Emberiza calandra* [NA: (33)]
- Corn crake – *Crex crex* [L: (36); N: (14, 36); NA: (47)]
- Dunnock – *Prunella modularis* [L: (11, 28, 39, 41, 43, 65, 70, 73, 76, 79, 105, 106, 109, 112, 113, 117, 120, 122, 128, 129, 133, 138, 142); N: (11, 28, 31, 34, 36, 39, 41, 43, 65, 70, 73, 76, 79, 82, 105, 106, 109, 111–113, 117, 119, 120, 122, 128, 129, 132, 133, 138, 142); NA: (57, 115, 145)]
- Eurasian blackcap – *Sylvia atricapilla* [L: (15, 17, 31, 34, 36, 39, 41, 44, 65, 70, 71, 73, 78, 93, 105, 112, 113, 116, 117, 119–122, 126, 128, 129, 131–133, 136, 138); N: (15, 28, 31, 34, 39, 41, 44, 65, 70, 73, 75, 80, 93, 105, 109, 111, 112^a^, 113, 116, 118, 119, 122, 126, 128, 129, 131–133, 138, 142); A: (118); NA: (33, 57, 115, 123, 143)]
- Eurasian blue tit- *Cyanistes caeruleus* [L: (13, 28, 34, 40, 105, 106, 116, 117, 120, 121, 129, 135, 137); N: (28, 34, 39, 40, 43, 73, 104–106, 117, 120, 126, 133); NA: (29, 33, 115, 123, 143, 145)]
- Eurasian bullfinch – *Pyrrhula pyrrhula* [L: (34, 39, 105, 117); N: (34, 36, 39, 105, 119, 131, 132, 138); NA: (115, 143)]
- Eurasian collared dove – *Streptopelia decaocto* [NA: (33)]
- Eurasian curlew – *Numenius arquata* [L: (107, 151); N: (107)]
- Eurasian eagle-owl – *Bubo bubo* (L: (152); N: (152, 153)]
- Eurasian golden oriole – *Oriolus oriolus* [L: (33); N: (33)]
- Eurasian hoopoe – *Upupa epops* [L: (93); N: (93, 108)]
- Eurasian jay – *Garrulus glandarius* [L: (15, 34, 44, 93, 116, 117, 129, 133); N: (14, 15, 33, 34, 43, 44, 70, 93, 116, 118, 129, 133); A: (118); NA: (26, 47, 115)]
- Eurasian magpie – *Pica pica* [L: (14); N: (14, 34); F: (14, 34); NA: (26, 30, 33, 35, 47)]
- Eurasian nuthatch – *Sitta europaea* [L:(15, 28, 34, 39, 116, 121, 129, 137); N: (34, 116, 121, 129, 133, 135); F: (52); NA: (33, 47, 48, 115, 123)]
- Eurasian oystercatcher – *Haematopus ostralegus* [L: (31); N: (31)]
- Eurasian reed warbler – *Acrocephalus scirpaceus* [L: (21, 28, 31, 39, 65, 112^a^, (128)]; N: (21, 25, 28, 31, 34, 43, 53, 65, 70, 73, 78, 104, 105, 109, 112^a^, 119, 128, 132, 142)]
- Eurasian siskin – *Carduelis spinus* [L: (24, 34, 39, 117); N: (34, 39, 70, 105, 117)]
- Eurasian sparrowhawk – *Accipiter nisus* [L: (24); N: (24, 31, 73, 105, 110, 140); A: (140)]
- Eurasian stonechat – *Saxicola torquatus* [NA: (30)]
- Eurasian tree sparrow – *Passer montanus* [L: (34, 70, 120); N: (34, 43, 70, 132); NA: (26, 33, 47)]
- Eurasian treecreeper – *Certhia familiaris* [L: (73, 117, 121, 122); N: (79, 104, 105, 122, 130)]
- Eurasian woodcock – *Scolopax rusticola* [N: (39); NA: (30, 33)]
- Eurasian wryneck – *Jynx torquilla* [NA: (33)]
- European crested tit – *Lophophanes cristatus* [L: (41); N: (41, 130)]
- European golden plover – *Pluvialis apricaria* [NA: (154)]
- European goldfinch – *Carduelis carduelis* [N: (109); NA: (33, 57)]
- European green woodpecker – *Picus viridis* [N: (133)]
- European greenfinch – *Carduelis chloris* [L: (15, 17, 105, 106, 112, 113, 117); N: (21, 33, 36, 39, 105, 106, 112, 113, 117, 120, 123, 142); NA: (46, 48, 57)]
- European herring gull – *Larus argentatus* [N: (43)]
- European honey buzzard – *Pernis apivorus* [NA: (155)]
- European pied flycatcher – *Ficedula hypoleuca* [L: (13, 21, 34, 36, 41); N: (34, 36, 70, 119); NA: (30, 48)]
- European robin – *Erithacus rubecula* [L: (11, 13–17, 28, 31, 34, 36, 39, 41, 43, 44, 65, 66, 70, 71, 73, 75, 76, 78, 79, 93, 105, 106, 109, 112^a^, 113, 116, 117, 119–122, 124, 126, 128–133, 135, 136, 138, 139, 141, 142, 156); N: (11, 13–17, 21, 28, 31, 34, 36, 39, 41, 43, 65, 66, 70, 71, 73, 78, 79, 82, 93, 104–106, 109–112^a^, 113, 116–122, 124, 127–133, 135, 136, 138–142, 149); F: (13, 39); A: (118); NA: (33, 48, 57, 73, 115, 123, 126, 143–145, 155)]
- European serin – *Serinus serinus* [L: (79); N: (17, 34, 79); NA: (33)]
- Fieldfare -*Turdus pilaris* [L: (14, 34, 75, 105, 112, 113); N: (14, 31, 34, 36, 74, 75, 105, 112^a^, 113); NA: (26, 47, 48, 57, 115)]
- Garden warbler – *Sylvia borin* [L: (36, 114, 119); N: (31, 34, 36, 70, 104, 105, 109, 112–114, 116, 119, 131); NA: (26, 33)]
- Goldcrest – *Regulus regulus* [L: (13, 31, 39, 41, 73); N: (13, 31, 39, 41, 73, 104, 105, 119, 141); NA: (145)]
- Golden eagle – *Aquila chrysaetos* [F: (34)]
- Great reed warbler – *Acrocephalus arundinaceus* [N: (104, 109, 112^a^, 140, 142)]
- Great spotted woodpecker – *Dendrocopos major* [L: (105, 106); N: (105, 106); NA: (33)]
- Great tit – *Parus major* [L: (13–15, 17, 21, 28, 31, 34, 36, 39–41, 43, 50, 51, 65, 70, 76, 78, 79, 105, 106, 116, 117, 120, 122, 124, 126, 129, 130, 132, 133, 135–137, 139, 140); N: (13–15, 17, 21, 25, 28, 34, 36, 39–41, 43, 50–52, 57, 65, 70, 73, 78, 79, 104–106, 112^a^, (116, 117, 120, 122, 124, 126, 129, 131, 133, 135–142, 146); M: (52); F: (52, 70); A: (124); NA: (29, 33, 115, 123, 143–145, 147, 157)]
- Greenish warbler – *Phylloscopus trochiloides* [L: (119); N: (36, 111)]
- Gray partridge – *Perdix perdix* [L: (31); N: (31, 108); NA: (14, 47)]
- Gray wagtail – *Motacilla cinerea* [L: (122); N: (57, 122)]
- Hawfinch – *Coccothraustes coccothraustes* [L: (33, 79, 121, 129, 135, 137, 139); N: (28, 33, 34, 65, 70, 79, 109, 112, 113, 121, 128, 129, 135– 137, 139, 140); A: (140); NA: (57)]
- Hazel grouse – *Tetrastes bonasia* [NA: (47)]
- Hooded crow – *Corvus cornix* [NA: (33, 47)]
- House sparrow – *Passer domesticus* [L: (70, 126, 151); N: (34, 70, 73); NA: (33, 47, 123)]
- Iberian chiffchaff – *Phylloscopus ibericus* [L: (15)]
- Icterine warbler – *Hippolais icterina* [L: (73, 105, 112, 113, 119); N: (34, 36, 70, 73, 112, 113, 132); NA: (48)]
- Lesser redpoll – *Carduelis cabaret* [L: (109); N: (70, 112, 113)]
- Lesser whitethroat – *Sylvia curruca* [L: (41, 73, 105, 106, 132); N: (31, 36, 41, 70, 73, 105, 106, 110–113, 132, 140)]
- Long-eared owl – *Asio otus* [N: (34); NA: (26, 30)]
- Long-tailed tit – *Aegithalos caudatus* [L: (78)]
- Mallard – *Anas platyrhynchos* [NA: (26)]
- Marsh tit – *Poecile palustris* [L: (116, 120, 129); N: (28, 36, 39, 116, 126, 129, 138); NA: (29, 33)]
- Marsh warbler – *Acrocephalus palustris* [L: (28, 31, 34, 132); N: (28, 31, 34, 70, 73, 104, 105, 109, 112^a^, 124, 131, 132, 140); NA: (143, 144)]
- Meadow pipit – *Anthus pratensis* [L: (24, 28, 31, 76, 107); N: (24, 31, 43, 70, 75, 107, 112, 113); M: (110); NA: (48)]
- Melodious warbler – *Hippolais polyglotta* [L: (71); N: (78)]
- Mew gull – *Larus canus* [L: (31); N: (31, 151)]
- Middle spotted woodpecker – *Dendrocoptes medius* (NA: (26, 33)]
- Mistle trush – *Turdus viscivorus* [L: (70, 79, 123, 133); N: (34, 79, 123, 133); NA: (30, 33, 47)]
- Northern goshawk – *Accipiter gentilis* [N: (108)]
- Northern lapwing – *Vanellus vanellus* [F: (43); NA: (30)]
- Oriental turtle dove – *Streptopelia orientalis* [N: (52)]
- Pechora pipit – *Anthus gustavi* [NA: (30)]
- Pied wheatear – *Oenanthe pleschanka* [NA: (30)]
- Red crossbill – *Loxia curvirostra* [N: (36); NA: (30)]
- Red kite – *Milvus milvus* [N: (34)]
- Red-backed shrike – *Lanius collurio* [L: (105); N: (73, 104, 105, 112^a^, 129); NA: (26, 33, 47)]
- Redwing – *Turdus iliacus* [L: (31, 36, 41, 65, 70, 73, 79, 105, 106, 109, 124, 136, 141); N: (31, 34, 36, 39, 41, 65, 70, 73, 76, 79, 104–106, 109, 111–114, 124, 128, 136, 141, 142); NA: (14, 48, 115, 144)]
- Ring ouzel – *Turdus torquatus* [N: (34, 138); NA: (30)]
- River warbler – *Locustella fluviatilis* [N: (129)]
- Rock bunting – *Emberiza cia* [N: (120)]
- Rook – *Corvus frugilegus* [L: (55); N: (52); M: (52); F: (52); NA: (26)]
- Rustic bunting – *Emberiza rustica* [NA: (26)]
- Sand martin – *Riparia riparia* [L: (95 in nest); N: (95 in nest)]
- Sardinian warbler – *Sylvia melanocephala* [L: (15); N: (21)]
- Savi's warbler –* Locustella luscinioides* [L: (65); N: (65, 142)]
- Sedge warbler – *Acrocephalus schoenobaenus* [L: (65, 128); N: (28, 65, 70, 104, 110, 112^a^, 128); NA: (30)]
- Short-toed treecreeper – *Certhia brachydactyla* [L: (15, 17, 34); N: (15, 17)]
- Skylark – *Alauda arvensis* [L: (105); N: (33, 70, 79); NA: (30)]
- Song thrush – *Turdus philomelos* [L: (11, 13–15, 24, 31, 34, 36, 39, 41, 44, 57, 65, 70, 73, 76, 78, 79, 93, 105, 106, 109, 112, 114, 116, 117, 119–122, 124, 126, 128, 130, 131, 133, 135–139, 141); N: (11, 13, 14, 16, 21, 24, 31, 34, 36, 39, 41, 44, 57, 65, 70, 73, 75, 76, 78, 79, 93, 104–106, 108, 109, 111–114, 116, 117, 119–122, 124, 126, 128, 130–133, 135–139, 141, 142, 146); NA: (33, 35, 115, 143, 144, 147)]
- Spotted flycatcher – *Muscicapa striata* [L: (110, 120, 132); N: (31, 34, 110)]
- Spotted nutcracker – *Nucifraga caryocatactes* (N: (79); NA: (26)]
- Tawny owl -*Strix aluco* [NA: (47)]
- Thrush nightingale – *Luscinia luscinia* [L: (16, 31, 36, 41, 65, 73, 105, 106, 112^a^, 119, 124, 128, 132, 138); N: (31, 36, 41, 65, 73, 105, 106, 112^a^, 119, 124, 128, 138, 140); A: (140); NA: (144)]
- Tree pipit – *Anthus trivialis* [L: (21, 31, 34, 36, 41, 43, 73, 79, 105, 106, 110, 121, 128, 132, 136); N: (24, 31, 34, 36, 41, 43, 73, 79, 104– 106, 110, 121, 128, 132, 136, 139); NA: (33, 35, 48)]
- Turkey – *Meleagris gallopavo* – [NA: (14)]
- Western jackdaw – *Corvus monedula* [L: (55); N: (55); NA: (33)]
- Western yellow wagtail – *Motacilla flava* [L: (34, 36); N: (31, 34, 110)]
- Wheatear – *Oenanthe oenanthe* [N: (31, 76, 79, 112, 113); NA: (59)]
- Whinchat – *Saxicola rubetra* [N: (112, 113, 138); NA: (30)]
- White wagtail – *Motacilla alba* [L: (73); N: (24, 52); M: (52)]
- Willow ptarmigan – *Lagopus lagopus* [L: (107, 158, 159); N: (107, 158, 159)]
- Willow tit – *Poecile montana* [L: (31, 39, 70, 120, 126); N: (31, 34, 39, 70, 112^a^, 120, 126, 130); NA: (26, 57)]
- Willow warbler – *Phylloscopus trochilus* [L: (11, 21, 31, 34, 36, 41, 43, 70, 73, 75, 79, 105, 106, 112, 113, 117, 119, 132, 138); N: (11, 28, 31, 34, 36, 41, 43, 70, 73, 75, 78–80, 104–106, 110–112^a^, 24, 113, 114, 138); NA: (33)]
- Winter wren – *Troglodytes troglodytes* [L: (15, 17, 21, 31, 34, 39, 41, 44, 65, 66, 70, 73, 78, 79, 105, 106, 114, 116, 117, 122, 129, 132, 133, 139, 141, 142); N: (15, 17, 21, 31, 34, 36, 39, 65, 66, 70, 73, 79, 104–106, 114, 116, 122–124, 129, 130, 133, 138–141); F: (25); NA: (33, 57, 73, 115, 143–145)]
- Wood warbler – *Phylloscopus sibilatrix* [L: (36, 41, 105, 106); N: (36, 41); NA: (33)]
- Woodlark – *Lullula arborea* [L: (112, 113); N: (79, 112, 113)]
- Yellow-browed warbler – *Phylloscopus inornatus* [NA: (48)]
- Yellowhammer – *Emberiza citrinella* [L: (33, 93); N: (33, 39, 93, 129); NA: (14, 26, 57)]

Distribution of reported cases: United Kingdom (24, 25, 30, 46, 53, 75, 76, 80, 107, 117, 150, 154, 158, 159), Sweden (31, 36, 56!, 73, 102!, 105, 106, 110, 119), Slovakia (33, 120, 126, 138), Poland (26, 39, 41, 51, 108, 121, 125, 135, 137), Switzerland (34, 79, 82, 115, 116, 118, 157), Latvia (130, 141), Germany (61!, 74, 124, 127, 131, 134, 143, 144), Czech Republic (28, 29, 57, 95!, 122, 129, 148, 149), Belgium (40, 48, 50, 152), Italy (22!, 78, 123, 136, 155, 156), Netherlands (43, 70, 151), Estonia (104), Norway (11, 109, 111–113), Hungary (47, 65, 128, 142), Faroe Islands (59), Denmark (132), Romania (13, 14, 23!, 55), France (133), Moldova (88!, 93), Russia (Kaliningrad, 114, 139, 147, 146), Ukraine (52), Portugal (15, 17, 21), Spain (44, 66, 71), Lithuania (112^a^, 145), Croatia (35), Bulgaria (140, 153), Cyprus (16).

#### Ixodes rothschildi

Overview: *Ixodes rothschildi* has so far been reported from birds only in the United Kingdom and France. This tick species is a parasite of seabirds, collected from 4 charadriiform, 1 suliform, and 1 procellariform species.

Charadriiformes: 4; Suliformes: 1; Procellariiformes: 1

Hosts:

- Atlantic puffin – *Fratercula arctica* [L: (76, 160); N: (76, 160); F: (76); NA: (18)]
- Common murre – *Uria aalge* [NA: (30)]
- European herring gull – *Larus argentatus* [NA: (30)]
- European shag – *Phalacrocorax aristotelis* [F: (75)]
- Manx shearwater – *Puffinus puffinus* [NA: (30)]
- Razorbill – *Alca torda* [N: (161); F: (161)in burrows]

Distribution of reported cases: United Kingdom (30, 75, 76, 160, 161), France (18).

#### Ixodes unicavatus

Overview: *Ixodes unicavatus* is distributed in the coastal area of the UK, Sweden and France. The hosts are birds that usually nest on rocky cliffs. Six of such bird species have been described as hosts of this parasite.

Charadriiformes: 1; Suliformes: 3; Passeriformes: 2; Falconiformes: 1

Hosts:

- Atlantic puffin – *Fratercula arctica* [NA: (30)]
- Common shag – *Gulosus aristotelis* [NA: (30)]
- European rock pipit – *Anthus petrosus* [NA: (30)]
- Great cormorant – *Phalacrocorax carbo* [L: (31); N: (31); F: (31); NA: (30)]
- Gyrfalcon – *Falco rusticolus* [NA: (30)]
- Water pipit – *Anthus spinoletta* [L: (24)]

Distribution of reported cases: United Kingdom (24, 30), Sweden (31), France (18!).

#### Ixodes uriae

Overview: *Ixodes uriae* is a common parasite of North European seabirds, as it was reported from 8 charadriiform, 1 anseriform, 3 suliform, and 1 procellariform bird species. According to the data it was also recorded from 2 passeriform bird species.

Charadriiformes: 8; Anseriformes: 1; Suliformes: 3; Procellariiformes: 1; Passeriformes: 3

Hosts:

- Atlantic puffin – *Fratercula arctica* [N: (24, 76, 162 in nest); M: (162 in nest); F: (24, 162 in nest); NA: (59, 163^a^, 163^b^); (164^a^, 164^b^, 165^a^, 166)]
- Black guillemot – *Cepphus grylle* [NA: (30)]
- Black-legged kittiwake – *Rissa tridactyla* (L: (167); N: (162 in nest, 167–170); M: (162 in nest, 167); F: (161, 162 in nest, 167, 169); NA: (163^b^, 164^a^, 164^b^, 165^a^, 170, 171^a^, 171^b^, 172, 173)]
- Common eider – *Somateria mollissima* [NA: (174)]
- Common murre – *Uria aalge* [N: (31, 162 in nest, 175–177); M: (162 in nest, 175); F: (31, 75, 161, 162 in nest, 175–177); NA: (59, 163^b^, 164^a^, 164^b^, 165^a^)]
- Common starling – *Sturnus vulgaris* [NA: (30)]
- Eurasian curlew – *Numenius arquata* [NA: (30)]
- European herring gull – *Larus argentatus* [N: (170 in nest); F: (170 in nest); NA: (30)]
- European shag – *Phalacrocorax aristotelis* [N: (76)]
- Northern fulmar – *Fulmarus glacialis* [NA: (30)]
- Northern gannet – *Morus bassanus* [N: (76); F: (76)]
- Razorbill – *Alca torda* [N: (177); F: (177); NA: (30, 163^b^, 164^a^, 164^b^)]
- Red crossbill – *Loxia curvirostra* [NA: (30)]
- Red-faced cormorant –* Urile urile* [NA: (163^b^)]
- Thick-billed murre – *Uria lomvia* [N: (175^a^); M: (175^a^); F: (175); NA: (178–180)]
- Wheatear – *Oenanthe oenanthe* [NA: (30)]

Distribution of reported cases: United Kingdom (24, 30, 76, 161, 163, 164, 167–169, 171, 172, 176), France (170, 171^a^), Norway (Svalbard) (175^a^, 178, 179), Norway (162, 163^a^, 164^a^, 165, 171^b^, 173, 177, 180), Norway (Jan Mayen) (175), Iceland (163^b^, 164^b^, 165, 174), Faeroe Islands (59, 166), Sweden (31).

#### Ixodes ventalloi

Overview: *Ixodes ventalloi*, commonly known as the rabbit tick as it mostly feeds on the European rabbit (*Oryctolagus cuniculus*), is a rare finding on birds (8). This tick species has been recorded from 11 bird species. Four species belong to the order Strigiformes, which are often in contact with small mammals.

Strigiformes: 4; Passeriformes: 3; Galliformes: 3, Gruiformes: 1

Hosts:

- Barn owl – *Tyto alba* [N: (60)]
- Black redstart – *Phoenicurus ochruros* [N: (84, 181)]
- Chukar partridge- *Alectoris chukar* [NA: (182)]
- Common blackbird – *Turdus merula* [N: (84); F: (21); NA: (25)]
- Common pheasant – *Phasianus colchicus* [A: (19)]
- European robin – *Erithacus rubecula* [N: (181)]
- Little owl – *Athene noctua* [F: (183)]
- Long-eared owl – *Asio otus* [N: (76); F: (76)]
- Red-legged partridge – *Alectoris rufa* [A: (19); NA: (90)]
- Short-eared owl – *Asio flammeus* [N: (85); M: (85); F: (85)]
- Water rail – *Rallus aquaticus* [A: (19)]

Distribution of reported cases: Cyprus (182), Spain (90, 183), Portugal (21, 60, 85), Italy (19, 84, 181), United Kingdom (25, 76).

### Metastriata

#### Amblyomma lepidum

Overview: While the immature stages of *Amlyomma lepidum* are relatively common bird parasites in Africa (10), this tick species has been reported only once in European territory, from a passeriform bird.

Passeriformes: 1

Hosts:

- Common blackbird – *Turdus merula* [N: (16)]

Distribution of reported cases: Cyprus (16).

#### Amblyomma marmoreum

Overview: *Amblyomma marmoreum* is an African tick species, where it was reported from several bird species (10). In Europe, it has been found on a Tree pipit in Italy.

Passeriformes: 1

Hosts:

- Tree pipit – *Anthus trivialis* [N: (84)]

Distribution of reported cases: Italy (84).

#### Amblyomma nuttalli

Overview: *Amlyomma nuttali* is distributed in the Afrotropical region, where they may infest several bird species (10). So far, we have only one report in Europe, where it was found on a Thrush nightingale (Passeriformes).

Passeriformes: 1

Hosts:

- Thrush nightingale – *Luscinia luscinia* [**N:** (16)]

Distribution of reported cases: Cyprus (16).

#### Amblyomma variegatum

Overview: *Amblyomma variegatum* is an African tick species (10) and as such, it does not have European distribution. However, it was reported from 2 passeriform birds in Italy, and from one passeriform in Cyprus.

Passeriformes: 2

Hosts:

- Icterine warbler – *Hyppolais icterina* [N: (184)]
- Tree pipit – *Anthus trivialis* [N: (16, 181)]

Distribution of reported cases: Italy (181, 184), Cyprus (16).

#### Dermacentor reticulatus

Overview: *Dermacentor reticulatus* has a pan European distribution excepting Scandinavia (8), however its occurrence on birds is extremely rare. The only reports are from the United Kingdom and from Poland.

Passeriformes: 2

Hosts:

- European robin – *Erithacus rubecula* [L: (39)]
- Meadow pipit – *Anthus pratensis* [N: (75)]

Distribution of reported cases: Poland (39), United Kingdom (75).

#### Dermacentor marginatus

Overview: *Dermacentor marginatus* is a common parasite throughout Europe, but not in Scandinavia (8). Despite this fact, it is rare to find these ticks on birds. *D. marginatus* has been reported from 1 passeriform and 1 galliform bird species, as well as from the nest of a passeriform bird.

Passeriformes: 2; Galliformes: 1

Hosts:

- Bearded reedling – *Panurus biarmicus* [L: (185 in nest); N: (185 in nest)]
- Turkey – *Meleagris gallopavo* – [A: (60)]
- Yellowhammer – *Emberiza citrinella* [NA: (14, 47)]

Distribution of reported cases: Austria (185), Hungary (47), Portugal (60), Romania (14).

#### Haemaphysalis concinna

**Overview:** *Haemaphysalis concinna* has a pan-European distribution. Interestingly, *H. concinna* seems to be extremely common on birds in Hungary, compared to other European countries. This parasite was reported from 36 different bird species so far. Thirty-three of these belong to the order Passeriformes.

Passeriformes: 33; Accipitriformes: 1; Galliformes: 1; Charadriiformes: 1

Hosts:

- Barred warbler – *Sylvia nisoria* [NA: (186)]
- Black kite – *Milvus migrans* [NA: (47)]
- Common blackbird – *Turdus merula* [L: (65); N: (52, 65, 129, 140); NA: (33, 186)]
- Common chaffinch – *Fringilla coelebs* [NA: (33)]
- Common grasshopper warbler – *Locustella naevia* [L: (65); N: (65)]
- Common nightingale – *Luscinia megarhynchos* [L: (65); N: (65); NA: (33, 186)]
- Common pheasant – *Phasianus colchicus* [NA: (33)]
- Common reed bunting – *Emberiza schoeniclus* (L: (65); N: (65)]
- Common starling – *Sturnus vulgaris* [NA: (33)]
- Dunnock – *Prunella modularis* [L: (65, 128, 129); N: (65); NA: (186)]
- Eurasian blackcap – *Sylvia atricapilla* [L: (17, 65, 128); N: (65, 128); NA: (186)]
- Eurasian blue tit- *Cyanistes caeruleus* [NA: (33)]
- Eurasian golden oriole – *Oriolus oriolus* (N: (33)]
- Eurasian magpie – *Pica pica* [NA: (33)]
- Eurasian nuthatch – *Sitta europaea* [NA: (33)]
- Eurasian reed warbler – *Acrocephalus scirpaceus* [L: (65, 128); N: (65, 128); NA: (186)]
- Eurasian tree sparrow – *Passer montanus* [NA: (33)]
- Eurasian woodcock – *Scolopax rusticola* [NA: (33)]
- European greenfinch – *Carduelis chloris* [L: (17); N: (65); NA: (186)]
- European robin – *Erithacus rubecula* [L: (65, 129); N: (65, 142); NA: (33, 186)]
- Great reed warbler – *Acrocephalus arundinaceus* [NA: (186)]
- Great tit – *Parus major* [L: (65); N: (52, 65); NA: (33, 186)]
- Hawfinch – *Coccothraustes coccothraustes* [L: (65); N: (65); NA: (14, 33, 186)]
- House sparrow – *Passer domesticus* [NA: (33)]
- Lesser whitethroat – *Sylvia curruca* [NA: (186)]
- Marsh warbler – *Acrocephalus palustris* [L: (65); N: (65); NA: (186)]
- Red-backed shrike – *Lanius collurio* [NA: (33, 186)]
- River warbler – *Locustella fluviatilis* [N: (129); NA: (186)]
- Rook – *Corvus frugilegus* [L: (55)]
- Savi's warbler –* Locustella luscinioides* [L: (65, 128); N: (65); NA: (186)]
- Sedge warbler –* Acrocephalus schoenobaenus* [L: (65); N: (65); NA: (186)]
- Song thrush – *Turdus philomelos* [L: (65); N: (65); NA: (33, 186)]
- Tree pipit – *Anthus trivialis* [NA: (33, 47)]
- Willow warbler – *Phylloscopus trochilus* [L: (16)]
- Winter wren – *Troglodytes troglodytes* [L: (129)]
- Yellowhammer – *Emberiza citrinella* [L: (33, 65, 129); N: (33, 65, 129); NA: (186)]

Distribution of reported cases: Czech Republic (57!, 129), Slovakia (33), Hungary (47, 65, 128, 142, 186), Romania (23!, 55), Ukraine (52), Bulgaria (140), Cyprus (16).

#### Haemaphyalis erinacei

Overview: *Haemaphysalis erinacei* (as its name suggests) is mainly a parasite of hedgehogs and small mammals (8). Finding this tick on birds is a rare event. So far, it has been reported from the South-Eastern European region, where *H. erinacei* was found on on 4 different bird species. Interestingly, these 4 birds belonged to 4 different orders.

Caprimulgiformes: 1; Strigiformes: 1, Gruiformes: 1, Coraciiformes: 1

Hosts:

- Alpine swift – *Apus melba* [N: (187^a^)]
- Common Crane – *Grus grus* [M: (187)]
- Eurasian eagle-owl – *Bubo bubo* [F: (153)]
- European Bee-eater – *Merops apiaster* [M: (187)]

Distribution of reported cases: Bulgaria (153), Croatia (187^a^), Bosnia and Herzegovina (187).

#### Haemaphyalis parva

Overview: *Haemaphysalis parva* is a rare tick species on European birds. In Romania, it was found on 2 passeriforms, and on 1 galliform bird.

Passeriformes: 2; Galliformes: 1

Hosts:

- Common snipe – *Gallinago gallinago* [NA: (14)]
- Rook – *Corvus frugilegus* [L: (55); N: (55)]
- Western jackdaw – *Corvus monedula* [L: (55)]

Distribution of reported cases: Romania (14, 55).

#### Haemaphysalis punctata

Overview: *Haemaphysalis punctata* has a pan-European distribution, and its immature stages are fairly common parasites on birds, especially on Passeriformes.

Passeriformes: 40; Charadriiformes: 7; Falconiformes: 1; Strigiformes: 1; Galliformes: 2

Hosts:

- Black-headed gull – *Chroicocephalus ridibundus* [F: (31); NA: (30)]
- Cirl bunting – *Emberiza cirlus* [L: (21, 66, 71); N: (21, 85)]
- Common blackbird – *Turdus merula* [L: (15, 17, 21, 66, 71, 72); N: (13, 15, 21, 66, 69, 71, 85, 188); NA: (30, 33, 46, 47)]
- Common chaffinch – *Fringilla coelebs* [L: (21, 66, 71); N: (69, 71, 188)]
- Common kestrel- *Falco tinnunculus* [NA: (30)]
- Common linnet – *Carduelis cannabina* [L: (73)]
- Common nightingale – *Luscinia megarhynchos* [N: (71)]
- Common redstart – *Phoenicurus phoenicurus* [L: (151); F: (31, 110); NA: (26)]
- Common reed bunting – *Emberiza schoeniclus* [NA: (30)]
- Common starling – *Sturnus vulgaris* [L: (73); NA: (30)]
- Common tern – *Sterna hirundo* [NA: (30)]
- Common whitethroat – *Sylvia communis* [L: (73); N: (31); NA: (30)]
- Dunnock – *Prunella modularis* [L: (66); N: (39, 66); NA: (30)]
- Eurasian blackcap – *Sylvia atricapilla* [N: (69)]
- Eurasian blue tit – *Cyanistes caeruleus* [L: (73)]
- Eurasian curlew – *Numenius arquata* [N: (151)]
- Eurasian eagle-owl – *Bubo bubo* [F: (153)]
- Eurasian jay – *Garrulus glandarius* [L: (15); NA: (17, 47)]
- Eurasian magpie – *Pica pica* [NA: (47)]
- Eurasian oystercatcher – *Haematopus ostralegus* [L: (31); N: (31)]
- Eurasian reed warbler – *Acrocephalus scirpaceus* [N: (85)]
- Eurasian stonechat – *Saxicola torquatus* [NA: (30)]
- Eurasian woodcock – *Scolopax rusticola* [NA: (33)]
- European greenfinch – *Carduelis chloris* [L: (71)]
- European herring gull – *Larus argentatus* [NA: (30)]
- European robin – *Erithacus rubecula* [L: (21, 66, 156); N: (66, 69); F: (140); NA: (30)]
- European serin – *Serinus serinus* [L: (71)]
- Great black-backed gull – *Larus marinus* [NA: (26)]
- Great tit – *Parus major* [L: (21, 66); N: (188)]
- Hooded crow – *Corvus cornix* [NA: (47)]
- House sparrow – *Passer domesticus* [L: (71, 151); N: (71, 151, 188); NA: (30)]
- Meadow pipit – *Anthus pratensis* [L: (151); NA: (30)]
- Melodious warbler – *Hippolais polyglotta* [NA: (30)]
- Mistle trush – *Turdus viscivorus* [NA: (30)]
- Red-billed chough – *Pyrrhocorax pyrrhocorax* (NA: (30)]
- Red-legged partridge – *Alectoris rufa* [NA: (87)]
- Ring ouzel – *Turdus torquatus* [NA: (47)]
- Rook – *Corvus frugilegus* [L: (55, 188); N: (55, 188); M: (55); F: (55); A: (188)]
- Skylark – *Alauda arvensis* [NA: (30)]
- Song thrush – *Turdus philomelos* [L: (93); N: (66, 93); NA: (30)]
- Spotless starling – *Sturnus unicolor* [L: (71); N: (71)]
- Spotted flycatcher – *Muscicapa striata* [L: (16)]
- Tree pipit – *Anthus trivialis* [L: (21)]
- Turkey – *Meleagris gallopavo* – [N: (60); A: (60); NA: (14)]
- Western jackdaw – *Corvus monedula* [N: (55); M: (55); NA: (30)]
- Western yellow wagtail – *Motacilla flava* [NA: (35)]
- Wheatear – *Oenanthe oenanthe* [L: (73); NA: (30)]
- Whinchat – *Saxicola rubetra* [L: (151)]
- White wagtail – *Motacilla alba* [L: (73); N: (31, 110); NA: (30)]
- Willow warbler – *Phylloscopus trochilus* [L: (16); NA: (30)]
- Yellowhammer – *Emberiza citrinella* [NA: (30, 33)]

Distribution of reported cases: Ukraine (188), Spain (66, 71, 87), Poland (26, 39), United Kingdom (30, 46), Slovakia (33), Netherlands (151), Sweden (31, 73, 110), Portugal (Azores, 69), Moldova (93), Portugal (15, 17, 21, 60, 85), Romania (13, 14, 55), Italy (156), Hungary (47), Croatia (35), Bulgaria (140, 153), Cyprus (16).

#### Haemaphyalis sulcata

Overview: *Haemaphysalis sulcata* is a relatively rare parasite on birds. According to literature data, it has been recorded from 5 passeriform bird species so far.

Passeriformes: 5

Hosts:

- Common blackbird – *Turdus merula* [N: (140)]
- Great reed warbler – *Acrocephalus arundinaceus* [N: (140)]
- Savi's warbler –* Locustella luscinioides* [N: (140)]
- Skylark – *Alauda arvensis* [N: (24)]
- Thrush nightingale – *Luscinia luscinia* [N: (140)]

Distribution of reported cases: United Kingdom (24), Bulgaria (140).

#### Hyalomma aegyptium

Hosts:

Overview: Although *Hyalomma aegyptium* is a common tick in the Mediterranean region, it is primarily a parasite of tortoises and humans (8). Therefore, finding these ticks on European birds is an uncommon event. The only European report of this tick feeding on birds so far is from Greece, where this parasite has been reported from 2 passeriform species.

Passeriformes: 2

- Common blackbird – *Turdus merula* [L: (20)]
- Common nightingale – *Luscinia megarhynchos* [L: (20)]

Distribution of reported cases: Greece (20).

#### Hyalomma lusitanicum

Overview: *Hyalomma lusitanicum* is a common parasite of mammals in Spain and Portugal (8, 10). Despite this fact, finding this tick species on bird is occasional. So far, it has been recorded from 2 galliform, 1 passeriform, 1 strigiform, 1 struthioniform and 1 columbiform birds from the Iberian Peninsula.

Passeriformes: 1; Galliformes: 2; Strigiformes: 1; Columbiformes: 1, Struthioniformes: 1

Hosts:

- Chicken – *Gallus gallus domesticus* [N: (189)]
- Common blackbird – *Turdus merula* [L: (15); N (15)]
- Common ostrich – *Struthio camelus* [A: (60)]
- Red-legged partridge – *Alectoris rufa* [N: (60); NA: (87)]
- Eurasian eagle-owl – *Bubo bubo* [N: (60)]
- Common wood pigeon – *Columba palumbus* [A: (60)]

Distribution of reported cases: Spain (87), Portugal (15, 60, 189).

#### Hyalomma marginatum (s.l.)

Overview: *Hyalomma marginatum* is a two-host tick species (10) that has a Palearctic distribution. While it is more common in the Mediterranean region, this parasite is also reported occasionally from Northern European countries. The most common bird hosts of *H. marginatum* are from the order Passeriformes.

Passeriformes: 52; Accipitriformes: 5; Galliformes: 1; Strigiformes: 5; Falconiformes: 2; Coraciiformes: 1; Bucerotiformes: 1; Caprimulgiformes: 1, Columbiformes: 1

Hosts:

- Barn owl – *Tyto alba* [L: (85); N: (85)]
- Barn swallow –* Hirundo rustica* [N: (85)]
- Black kite – *Milvus migrans* [N: (85)]
- Black redstart – *Phoenicurus ochruros* [N: (16)]
- Bluethroat – *Luscinia svecica* [N: (190, 191); NA: (191^b^)]
- Booted eagle – *Hieraaetus pennatus* [N: (60)]
- Chicken – *Gallus gallus domesticus* [NA: (14)]
- Collared flycatcher – *Ficedula albicollis* [L: (31)]
- Common blackbird – *Turdus merula* [L: (15, 71); N: (16, 71, 85, 192)]
- Common buzzard – *Buteo buteo* [N: (60)]
- Common chaffinch – *Fringilla coelebs* [L: (16, 71); N: (16); F: (73)]
- Common chiffchaff – *Phylloscopus collybita* [L: (16)]
- Common kestrel – *Falco tinnunculus* [L: (60); N: (60); NA: (191^d^)]
- Common kingfisher – *Alcedo atthis* [N: (60, 85)]
- Common nightingale – *Luscinia megarhynchos* [L: (71, 193^a^); N: (71, 140, 191^e^, 193^a^)]
- Common redstart – *Phoenicurus phoenicurus* [N: (16, 80, 82, 184, 190, 191^c^); NA: (30, 191^b^, 191^d^)]
- Common whitethroat – *Sylvia communis* [N: (80, 140, 191^c^, 194); NA: (30)]
- Corn bunting – *Emberiza calandra* [L: (16)]
- Crested lark – *Galerida cristata* [L: (16); N: (16)]
- Dunnock – *Prunella modularis* [N: (195)]
- Eastern olivaceous warbler – *Iduna pallida* [N: (20)]
- Eastern woodchat shrike – *Lanius senator niloticus* [N: (196)]
- Eurasian blackcap – *Sylvia atricapilla* [L: (31); N: (16, 191^c^)]
- Eurasian blue tit- *Cyanistes caeruleus* [N: (71, 85)]
- Eurasian eagle-owl – *Bubo bubo* [L: (153); N: (60, 85, 153); M: (153)]
- Eurasian hoopoe – *Upupa epops* [NA: (191^d^)]
- Eurasian jay – *Garrulus glandarius* [N: (15)]
- Eurasian reed warbler – *Acrocephalus scirpaceus* [L: (193); N: (60, 85); M: (190); F: (190, 193^a^); NA: (38)]
- Eurasian scops owl – *Otus scops* [L: (16); N: (16)]
- Eurasian stonechat – *Saxicola torquatus* [L: (16); N: (16, 60, 85)]
- Eurasian tree sparrow – *Passer montanus* [N: (85)]
- European greenfinch – *Carduelis chloris* [L: (16); N: (16, 71, 85)]
- European nightjar – *Caprimulgus europaeus* [N: (184)]
- European pied flycatcher – *Ficedula hypoleuca* [L: (71)]
- European robin – *Erithacus rubecula* [L: (73, 142); N: (16, 82, 142, 191^a^, 195)]
- European turtle dove – *Streptopelia turtur* [L: (16)]
- Finsch's wheatear – *Oenanthe finschii* [L: (16); N: (16)]
- Great reed warbler – *Acrocephalus arundinaceus* [L: (16); N: (193^a^)]
- Great tit – *Parus major* [L: (71); N: (71, 85)]
- House sparrow – *Passer domesticus* [L: (71); N: (71)]
- Iberian gray shrike – *Lanius meridionalis* [N: (85)]
- Lesser kestrel – *Falco naumanni* [N: (60)]
- Lesser whitethroat – *Sylvia curruca* (L: (16)]
- Little owl – *Athene noctua* [N: (85); NA: (14)]
- Marsh warbler – *Acrocephalus palustris* [L: (191^a^); N: (16, 140, 191^a^, 191^c^); NA: (193^a^)]
- Mistle trush – *Turdus viscivorus* [N: (190, 192)]
- Northern goshawk – *Accipiter gentilis* [N: (60)]
- Ortolan bunting – *Emberiza hortulana* [N: (191^b^)]
- Red kite – *Milvus milvus* [L: (85)]
- Rook – *Corvus frugilegus* [N: (192)]
- Sardinian warbler – *Sylvia melanocephala* [L: (16); N: (16)]
- Savi's warbler –* Locustella luscinioides* [N: (193)]
- Sedge warbler –* Acrocephalus schoenobaenus* [N: (31, 80, 193, 194, 196); F: (193); NA: (30, 102)]
- Song thrush – *Turdus philomelos* [L: (16); N: (16, 197)]
- Spanish Sparrow – *Passer hispaniolensis* [L: (16)]
- Spotted flycatcher – *Muscicapa striata* [L: (16); N: (14)]
- Tawny owl -*Strix aluco* [N: (60)]
- Thrush nightingale – *Luscinia luscinia* [L: (16)]
- Tree pipit – *Anthus trivialis* [L: (16); N: (16, 36, 191^b^)]
- Water pipit – *Anthus spinoletta* [L: (16)]
- Western jackdaw – *Corvus monedula* [N: (55)]
- Western olivaceous warbler – *Iduna opaca* [N: (194)]
- Western yellow wagtail – *Motacilla flava* [L: (16, 196); N: (16, 184); NA: (102)]
- Wheatear – *Oenanthe oenanthe* [N: (80); NA: (30)]
- Whinchat – *Saxicola rubetra* [N: (16); NA: (30)]
- White wagtail – *Motacilla alba* [L: (16); N: (16, 31, 190)]
- Willow warbler – *Phylloscopus trochilus* [L: (16, 194); N: (73)]
- Woodchat shrike – *Lanius senator* [L: (198); N: (198)]
- Yellowhammer – *Emberiza citrinella* [NA: (14)]

Distribution of reported cases: Switzerland (82, 190), Sweden (31, 36, 56!, 73), Slovakia (191^a^, 193), Czech Republic (191, 193^a^), Italy (184, 191^e^, 199!), Greece (20, 196, 198), United Kingdom (30, 80), Hungary (142), Romania (14, 55, 197), Portugal (15, 60, 85), Spain (71, 194), France (18!, 195), Poland (102), Germany (38, 191^c^), Finland (191^b^), Slovenia (191^d^), Ukraine (192), Bulgaria (140, 153), Cyprus (16).

#### Hyalomma rufipes

Overview: *Hyalomma rufipes* is a two-host tick species (8). It is a widely distributed, common bird parasite in the Mediterranean region. It isc occasionally reported on migrating birds from Northern European countries as well. Similarly to *H. marginatum* [in the past, *H rufipes* was considered as a subspecies of *H. marginatum* (10)] the most common bird hosts belong to Passeriformes.

Passeriformes: 46; Cuculiformes: 1; Piciformes: 1; Columbiformes: 1, Flaconiformes: 1, Bucerotiformes: 1, Strigiformes: 1

Hosts:

- Barred warbler – *Sylvia nisoria* [N: (16)]
- Barn swallow –* Hirundo rustica* [M: (200)]
- Black redstart – *Phoenicurus ochruros* [N: (181); NA: (84)]
- Black-eared Wheatear – *Oenanthe hispanica* [L: (16); N: (16)
- Blue rock thrush – *Monticola solitarius* [L: (16)]
- Crested lark – *Galerida cristata* [L: (16); N: (16)]
- Cyprus warbler – *Sylvia melanothorax* [N: (16)]
- Collared flycatcher – *Ficedula albicollis* [N: (16, 181, 184); NA: (84)]
- Common blackbird – *Turdus merula* [L: (16); N: (16)]
- Common chaffinch – *Fringilla coelebs* [N: (16)]
- Common chiffchaff – *Phylloscopus collybita* [L: (16)]
- Common cuckoo – *Cuculus canorus* [N: (16, 184)]
- Common kestrel- *Falco tinnunculus* [L: (16); N: (16)]
- Common nightingale – *Luscinia megarhynchos* [L: (184); N: (16, 184, 201)]
- Common redstart – *Phoenicurus phoenicurus* [L: (16, 184); N: (11, 16, 181, 184); NA: (84)]
- Common whitethroat – *Sylvia communis* [L: (184); N: (11, 16, 65, 181, 184); NA: (84)]
- Corn bunting – *Emberiza calandra* [L: (16)]
- Cretzschmar's bunting – *Emberiza caesia* [L: (16)]
- Eastern olivaceous warbler – *Iduna pallida* [L: (16)]
- Eastern subalpine warbler – *Sylvia cantillans* [L: (184)]
- Eurasian blackcap – *Sylvia atricapilla* [L: (16); N: (16, 195)]
- Eurasian golden oriole – *Oriolus oriolus* [N: (181, 184)]
- Eurasian hoopoe – *Upupa epops* [N: (16)]
- Eurasian reed warbler – *Acrocephalus scirpaceus* [L: (184); N: (11)]
- Eurasian scops owl – *Otus scops* [L: (16); N: (16)]
- Eurasian stonechat – *Saxicola torquatus* [L: (16); N: (16)]
- Eurasian wryneck – *Jynx torquilla* [L: (184)]
- European goldfinch – *Carduelis carduelis* [N: (16)]
- European greenfinch – *Carduelis chloris* [N: (16)]
- European pied flycatcher – *Ficedula hypoleuca* [L: (184), N: (184)]
- European robin – *Erithacus rubecula* [N: (16); NA: (84)]
- European turtle dove – *Streptopelia turtur* [L: (184)]
- Garden warbler – *Sylvia borin* [L: (184); N: (11)]
- Great reed warbler – *Acrocephalus arundinaceus* [L: (184); N: (184)]
- Icterine warbler – *Hyppolais icterina* [L: (184); N: (184)]
- Isabelline wheatear – *Oenanthe isabellina* [N: (16)]
- Lesser whitethroat – *Sylvia curruca* [L: (16); N: (16)]
- Pied wheatear – *Oenanthe pleschanka* [L: (16); N: (16)]
- Red-backed shrike – *Lanius collurio* [L: (16); N: (16)]
- Red-throated Pipit – *Anthus cervinus* [L: (16); N: (16)]
- Rock thrush – *Monticola saxatilis* [L: (16); N: (16)]
- Sedge warbler – *Acrocephalus schoenobaenus* [N: (184, 200); M: (200); F: (200)]
- Song thrush – *Turdus philomelos* [L: (16); N: (16); NA: (84)]
- Spotted flycatcher – *Muscicapa striata* [L: (184); NA: (84)]
- Thrush nightingale – *Luscinia luscinia* [N: (11)]
- Tree pipit – *Anthus trivialis* [L: (16, 181); N: (16, 184)]
- Orphean Warbler – *Sylvia hortensis* [L: (16)]
- Western yellow wagtail – *Motacilla flava* [L: (184, 191^f^)]
- Wheatear – *Oenanthe oenanthe* [L: (191^f^, 16), N: (11, 16, 181, 184); NA: (84)]
- Whinchat – *Saxicola rubetra* [L: (184); N: (181, 184, 202); NA: (84)]
- Willow warbler – *Phylloscopus trochilus* [L: (184); N: (16)]
- Wood warbler – *Phylloscopus sibilatrix* [L: (184); N: (184); NA: (84)]

Distribution of reported cases: Norway (11), Hungary (65), Italy (84, 181, 184, 202), France (Corsica, 200), France (195, 201), Finland (191^f^), Cyprus (16).

#### Hyalomma truncatum

Overview: There is very limited information about *Hyalomma truncatum* as a bird parasite. According to the data, it has been found on a passeriform bird in Italy.

Passeriformes: 1

Hosts:

- Whinchat – *Saxicola rubetra* [L: (184)]

Distribution of reported cases: Italy (184).

#### Rhipicephalus annulatus

Overview: Despite the fact that *Rhipicephalus annulatus* is distributed in Southern-Europe (8) it is amazingly rare for this parasite to feed on birds. So far, only one such case was documented in Portugal.

Galliformes: 1

Hosts:

- Turkey – *Meleagris gallopavo* – [N: (60); A: (60)]

Distribution of reported cases: Portugal (60).

#### Rhipicephalus pusillus

Overview: *Rhipicephalus pusillus* is found in the Palaearctic region, mainly in Mediterranean climatic areas where birds are considered to be exceptional hosts (8). So far, this was recorded only once.

Strigiformes: 1

Hosts:

- Eurasian eagle-owl – *Bubo bubo* [A: (60)]

Distribution of reported cases: Portugal (60).

#### Rhipicephalus sanguineus

Overview: *Rhipicephalus sanguineus s.l*. is the most common tick species found on dogs in urban areas around the world (8). However, finding them on birds is rare. So far, it has been recorded from a total of 6 bird species, belonging to 4 different orders.

Struthioniformes: 1; Strigiformes: 1; Passeriformes: 2; Accipitriformes: 2

Hosts:

- Common ostrich – *Struthio camelus* [A: (60); NA: (30)]
- Eurasian eagle-owl – *Bubo bubo* [A: (60)]
- Great reed warbler – *Acrocephalus arundinaceus* [F: (14)]
- House sparrow – *Passer domesticus* [F: (201) *Rhipicephalus sanguineus* sensu stricto (based on 99.7% 12S rRNA gene sequence identity between AY559843 and MH630345).
- Northern goshawk – *Accipiter gentilis* [A: (60)]
- Short-toed snake eagle – *Circaetus gallicus* [A: (60)]

Distribution of reported cases: Romania (14), France (201), Portugal (60), United Kingdom (30).

#### Rhipicephalus turanicus

Overview: The taxonomic status of *Rhipicephalus turanicus* is still under discussion (8). According to literature data, this tick species has been found on three bird species (all birds of prey) in Portugal and in Bulgaria. However, the Portuguese findings (85) were referred to in a later article by the same author (60) as *R. sanguineus*.

Accipitriformes: 2; Strigiformes: 1

Hosts:

- Common buzzard – *Buteo buteo* [F: (85)] [In a later article (60), the same author referred to this finding as *R. sanguineus*]
- Eurasian eagle-owl – *Bubo bubo* [M: (153); F: (153)]
- Steppe eagle – *Aquila nipalensis* [F: (85)] [In a later article (60), the same author referred to this finding as *R. sanguineus*]

Distribution of reported cases: Portugal (85), Bulgaria (153).

## Conclusions

In this review data on the ixodid tick infestation of birds were collected from nearly 200 papers published since 1952. In this period, 37 hard tick species (17 from Prostriata and 20 from Metastriata) were reported from 16 orders of avian hosts in Europe. These include endophilic tick species that are ornithophilic (*I. arboricola, I. caledonicus*) or those that prefer mammalian hosts, such as rodents (*I. acuminatus*), carnivores, or insectivores (*I. canisuga, I. hexagonus*). Some of the tick species have a clear host preference for sea birds and predominate in Western-Northern Europe (*I. rothschildi, I. unicavatus, I. uriae*). A significant number of ixodid species are rarely found in association with birds, as exemplified by *D. reticulatus, D. marginatus, H. erinacei, H. parva, H. sulcata, R. annulatus, R. turanicus, R. sanguineus*, and *H. aegyptium*. The latter is a tortoise-associated tick species, whereas *R. turanicus* and *R. sanguineus* are mostly reported from birds of prey, most likely transferring from rodent and other prey items to these birds. In addition, exotic (non-indigenous) tick species transported by birds from Africa to Europe include *H. truncatum, A. lepidum, A. marmoreum, A. nuttalli* and *A. variegatum*.

The primary aim of this checklist is to provide a comprehensive reference source (baseline data) for future studies, particularly in the context of discovering new tick-host associations after comparison with already published data. Nevertheless, these data also allow a first-hand analysis of general trends regarding how and which developmental stage of ticks tend to infest avian hosts in general. Therefore, five tick species that were frequently reported from birds and show a broad geographical distribution on this continent were selected for statistical comparison (*I. arboricola, I. frontalis, I. ricinus, H. concinna* and *H. marginatum*) (Supplementary Figure 1; Supplementary Table 1).

Considering developmental stages, larvae and nymphs predominate on birds in the case of *I. ricinus, H. concinna*, and *H. marginatum* (*n* = 1,667 vs. 37 adults). This is not the case for ornithophilic tick species (*I. arboricola* and *I. frontalis*) from which adult ticks were collected significantly more frequently (*n* = 301 vs. 115 adults) (*P* < 0.0001). Interestingly, males of generalist tick species occurred significantly more frequently on birds (*n* = 7 vs. 30 females) than males of ornithophilic tick species (*n* = 5 vs. 110 females) (*P* = 0.009).

Based on data that did not specify the tick developmental stage, the tick species *I. arboricola* was significantly (*P* < 0.0001) more frequently collected from bird species that typically feed above the ground (*n* = 134) than from those feeding at the ground level (*n* = 60). This was not the case for *I. ricinus* (679 vs. 1,064), *I. frontalis* (121 vs. 216) and *H. concinna* (36 vs. 63) (Supplementary Table 1). This general tendency on a continental level is in contrast to what was reported from the southern part of Central Europe where *H. concinna* is mostly associated with bird species typically feeding above the ground level (65, 127). The chances for finding *H. marginatum* on bird species characterized by either ground-feeding or arboreal feeding were more equilibrated (82 vs. 84). This is in line with its active host seeking (hunting) strategy (8).

In addition, considering data from references that included the number of tick developmental stages collected from various bird species, comparison of tick infestations according to five habitat categories of avian hosts (Supplementary Table 1) did not reveal statistically significant associations (data not shown). However, by assigning bird species into two groups (i.e., typically feeding from the ground vs. rarely occurring at the soil level: Supplementary Table 1), the following differences were found: Larvae, nymphs and females of *I. arboricola* significantly more frequently occur on bird species feeding above the ground (*P* < 0.0001-0.045), than corresponding stages of *I. frontalis, I. ricinus* and *H. concinna*. Females of *I. ricinus* are more likely to infest ground-feeding than arboreal bird species (18 vs. 4) compared to nymphs of this species (507 vs. 336) (*P* = 0.046). Interestingly, females of *H. marginatum* significantly more frequently associate with bird species feeding above the ground level than with those collecting food on the soil surface (4 vs. 1) compared to those of *I. ricinus* (4 vs. 18) (*P* = 0.017) and *I. frontalis* (27 vs. 61) (*P* = 0.041).

The ecology of bird-infesting tick species (8) is also illustrated here according to avian orders (Supplementary Figure 1), taking into account the ecology (habitat type) and activity (circadian rhythm and feeding level) of most bird species that belong to a certain order (203) (though this has limitations due to difficulties in assigning general traits at this taxonomic level). In this context, *I. arboricola* was only reported from species of avian orders whose members typically use forested habitats and (with the exception of Columbiformes) typically feed above the ground (Supplementary Figure 1). This tick species was also reported from a high number of nocturnal bird species (Strigiformes), although (as an endophilic tick, with preference for tree holes) it is known to detach from diurnal passerine hosts during the night (50). Except for two bird species from two orders (Falconiformes, Coraciiformes), *I. frontalis* was always reported from avian orders whose members typically (also) feed from the ground level, in both open and forested habitats (Supplementary Figure 1). This is in line with previous data on *I. frontalis* from Central Europe (65). By contrast, *I. ricinus* was reported from 13 out of 16 avian orders, including several examples with preference for forest or open habitats, and which typically feed at the ground level or higher (Supplementary Figure 1). Similarly, the majority of avian hosts that were reported to be infested with *H. concinna* represent orders that share both open habitat- and forest-dwelling bird species, as well as ground level and arboreal feeders (Supplementary Figure 1). Last but not least, *H. marginatum* was almost exclusively reported from bird species that belong to orders including a significant number of forest dweller bird species and was also collected from a high number of bird species from orders with nocturnal activity (both Caprimulgiformes and Strigiformes: Supplementary Figure 1). This is in agreement with the reported evening activity and occurrence of this tick species in forested habitats (204).

## Data availability statement

The original contributions presented in the study are included in the article/Supplementary material, further inquiries can be directed to the corresponding author/s.

## Ethics statement

Ethical review and approval was not required for the animal study because the study is based on a bibliographic review, no physical animals were handled.

## Author contributions

GK: conceptualization, study design, data curation, and manuscript writing. AS: study design, statistical analyses, ornithological data, and collection of literature data. SH: conceptualization, study design, editing, statistical analyses, and manuscript writing. All authors contributed to the article and approved the submitted version.

## Funding

This study was funded by Project no. TKP2020-NKA-01 implemented with the support provided from the National Research, Development and Innovation Fund of Hungary, financed under the Tématerületi Kiválósági Program 2020 (2020-4.1.1-TKP2020) funding scheme. SH and AS were supported by OTKA K-132794 of the National Research, Development and Innovation Office. The Project is supported by the European Union and co-financed by the European Social Fund (grant agreement no. EFOP-3.6.3-VEKOP-16-2017-00005, project title: Strengthening the scientific replacement by supporting the academic workshops and programs of students, developing a mentoring process).

## Conflict of interest

The authors declare that the research was conducted in the absence of any commercial or financial relationships that could be construed as a potential conflict of interest.

## Publisher's note

All claims expressed in this article are solely those of the authors and do not necessarily represent those of their affiliated organizations, or those of the publisher, the editors and the reviewers. Any product that may be evaluated in this article, or claim that may be made by its manufacturer, is not guaranteed or endorsed by the publisher.

## Supplementary material

Supplementary Figure 1 Reported occurrence of tick species according to avian orders. The number of bird species within an avian order, from which the relevant tick species was reported is encircled next to the name of avian order, along the line which connects it to the relevant tick species. Grouping of tick species according to habit preference, and of avian orders according to habitat type, nocturnal activity (inverse characters) and typical feeding level is simplified according to predominant traits of species in Europe (8, 203).

Supplementary Table 1 List of bird host species and tick-occurrence cases extracted from publications. Bird species in red are rare vagrants or captive populations, they do not occur regularly in Europe.

## References

[1] Jongejan F Uilenberg G. The global importance of ticks. Parasitology. (2004) 129:S3–14. 10.1017/S0031182004005967 15938502

[2] Rocklöv J Dubrow R. Climate change: an enduring challenge for vector-borne disease prevention and control. Nat Immunol. (2020) 21:479–83. 10.1038/s41590-020-0648-y 32313242 PMC7223823

[3] Rochlin I Toledo A. Emerging tick-borne pathogens of public health importance: a mini-review. J Med Microbiol. (2020) 69:781–91. 10.1099/jmm.0.001206 32478654 PMC7451033

[4] Hornok S Karcza Z Csörgo T. Birds as disseminators of ixodid ticks and tick-borne pathogens: note on the relevance to migratory routes. Ornis Hung. (2012) 20:86–9. 10.2478/orhu-2013-0010

[5] Knight MM Norval RA Rechav Y. The life cycle of the tick *Hyalomma marginatum rufipes* Koch (Acarina: Ixodidae) under laboratory conditions. J Parasitol. (1978) 64:143–6. 10.2307/3279627 627955

[6] Hornok S Horváth G. First report of adult *Hyalomma marginatum rufipes* (vector of Crimean-Congo haemorrhagic fever virus) on cattle under a continental climate in Hungary. Parasit Vectors. (2012) 5:170. 10.1186/1756-3305-5-170 22889105 PMC3436687

[7] Grandi G Chitimia-Dobler L Choklikitumnuey P Strube C Springer A Albihn A. First records of adult *Hyalomma marginatum* and *H. rufipes* ticks (Acari: Ixodidae) in Sweden. Ticks Tick-Borne Dis. (2020) 11:101403. 10.1016/j.ttbdis.2020.101403 32037097

[8] Estrada-Peña A Mihalca AD Petney TN. Ticks of Europe and North Africa: A Guide to Species Identification. Cham: Springer (2018). 10.1007/978-3-319-63760-0

[9] Gilot B Beaucournu JC Chastel C. Collecte ≪ au drapeau ≫ et fixation sur l'homme d'*Ixodes (Trichotoixodes) frontalis* (Panzer, 1795). Parasite. (1997) 4:197–9. 10.1051/parasite/1997042197 9296062

[10] Guglielmone AA Robbins RG Apanaskevich DA Petney TN Estrada-Peña A Horak IG. The Hard Ticks of the World: (Acari: Ixodida: Ixodidae). Dordrecht: Springer Science & Business Media. (2013). 10.1007/978-94-007-7497-1

[11] Hasle G Bjune G Edvardsen E Jakobsen C Linnehol B Røer JE. Transport of ticks by migratory passerine birds to Norway. J Parasitol. (2009) 95:1342–51. 10.1645/GE-2146.1 19658452

[12] Kuhl H Frankl-Vilches C Bakker A Mayr G Nikolaus G Boerno ST. An unbiased molecular approach using 3'-UTRs resolves the avian family-level Tree of Life. Mol Biol Evol. (2021) 38:108–27. 10.1093/molbev/msaa191 32781465 PMC7783168

[13] Sándor AD Mărcutan DI D'Amico G Gherman CM Dumitrache MO Mihalca AD. Do the ticks of birds at an important migratory hotspot reflect the seasonal dynamics of *Ixodes ricinus* at the migration initiation site? A case study in the Danube Delta. PLoS ONE. (2014) 9:e89378. 10.1371/journal.pone.0089378 24586732 PMC3929702

[14] Mihalca AD Dumitrache MO Magdaş C Gherman CM Domşa C Mircean V. Synopsis of the hard ticks (Acari: Ixodidae) of Romania with update on host associations and geographical distribution. Exp Appl Acarol. (2012) 58:183–206. 10.1007/s10493-012-9566-5 22544174

[15] Norte AC Ramos JA Gern L Núncio MS Lopes de Carvalho I. Birds as reservoirs for *Borrelia burgdorferi* s.l. *i*n Western Europe: circulation of *B. turdi* and other genospecies in bird-tick cycles in Portugal. Environ Microbiol. (2013) 15:386–97. 10.1111/j.1462-2920.2012.02834.x 22882497

[16] Kaiser MN Hoogstraal H Watson GE. Ticks (Ixodoidea) on migrating birds in Cyprus, fall 1967 and spring 1968, and epidemiological considerations. Bull Entomol Res. (1974) 64:97–110. 10.1017/S0007485300027024

[17] Norte AC de Carvalho IL Ramos JA Gonçalves M Gern L Núncio MS. Diversity and seasonal patterns of ticks parasitizing wild birds in western Portugal. Exp Appl Acarol. (2012) 58:327–39. 10.1007/s10493-012-9583-4 22669280

[18] Rageau J. Répartition géographique et rôle pathogène des tiques (acariens: Argasidae et Ixodidae) en France. Wiad Parazytol. (1972) 18:707–19. 4660775

[19] Tomassone L Grego E Auricchio D Iori A Giannini F Rambozzi L. Lyme borreliosis spirochetes and spotted fever group rickettsiae in ixodid ticks from Pianosa island, Tuscany Archipelago, Italy. Vector Borne Zoonotic Dis Larchmt N. (2013) 13:84–91. 10.1089/vbz.2012.1046 23289398

[20] Diakou A Norte AC Lopes de Carvalho I Núncio S Nováková M Kautman M. Ticks and tick-borne pathogens in wild birds in Greece. Parasitol Res. (2016) 115:2011–6. 10.1007/s00436-016-4943-3 26847630

[21] Norte AC Da Silva LP Tenreiro PJQ Felgueiras MS Araújo PM Lopes PB. Patterns of tick infestation and their *Borrelia burgdorferi* sl infection in wild birds in Portugal. Ticks Tick-Borne Dis. (2015) 6:743–50. 10.1016/j.ttbdis.2015.06.010 26159798

[22] Llopis IV Tomassone L Grego E Silvano F Rossi L. Investigation into Usutu and West Nile viruses in ticks from wild birds in Northwestern Italy, 2012-2014. New Microbiol. (2017) 40:56–7. 27819397

[23] Mărcutan I-D Kalmár Z Ionică AM D'Amico G Mihalca AD Vasile C. Spotted fever group rickettsiae in ticks of migratory birds in Romania. Parasit Vectors. (2016) 9:294. 10.1186/s13071-016-1565-7 27207258 PMC4875720

[24] Arthur DR Thompson GB. LXXIX.—Records of ticks collected from birds in the British Isles. J Nat Hist. (1953) 6:797–800. 10.1080/00222935308654485

[25] Jameson LJ Medlock JM. Tick surveillance in Great Britain. Vector Borne Zoonotic Dis. (2011) 11:403–12. 10.1089/vbz.2010.0079 20849277

[26] Siuda K Majszyk A Nowak M. Ticks (Acari: Ixodida) parasitizing birds (Aves) in Poland. Biol Lett. (2006) 43:147–51.

[27] Kristofěk J MaSÁN P Sustek Z Kloubec B. Arthropods (Pseudoscorpionida, Acari, Coleóptera, Siphonaptera) in nests of the tengmalm's owl, *Aegolius funereus*. Biol Bratisl. (2003) 58:231–40.

[28] Spitalská E Literák I Kocianová E Taragel'ová V. The importance of *Ixodes arboricola* in transmission of *Rickettsia* spp., *Anaplasma phagocytophilum*, and *Borrelia burgdorferi* sensu lato in the Czech Republic, Central Europe. Vector Borne Zoonotic Dis Larchmt N. (2011) 11:1235–41. 10.1089/vbz.2010.0210 21612531

[29] Literak I Kocianova E Dusbabek F Martinu J Podzemny P Sychra O. Winter infestation of wild birds by ticks and chiggers (Acari: Ixodidae, Trombiculidae) in the Czech Republic. Parasitol Res. (2007) 101:1709–11. 10.1007/s00436-007-0702-9 17674046

[30] Martyn KP. Provisional atlas of the ticks (Ixodoidea) of the British Isles. Biological Records Centre. (1988).

[31] Jaenson TG Tälleklint L Lundqvist L Olsen B Chirico J Mejlon H. Geographical distribution, host associations, and vector roles of ticks (Acari: Ixodidae, Argasidae) in Sweden. J Med Entomol. (1994) 31:240–56. 10.1093/jmedent/31.2.240 8189415 PMC7107449

[32] Novakova M Bulkova A Costa FB Kristin A Krist M Krause F. Molecular characterization of “*Candidatus* Rickettsia vini” in *Ixodes arboricola* from the Czech Republic and Slovakia. Ticks Tick-Borne Dis. (2015) 6:330–3. 10.1016/j.ttbdis.2015.02.006 25769386

[33] Ferianc O Lichard M. Birds in the Tribec and Hronský Inovec mountains as hosts of ticks. Bull World Health Organ. (1967) 36:19–23. 5298538 PMC2476104

[34] Papadopoulos B Humair PF Aeschlimann A Vaucher C Buttiker W. Ticks on birds in Switzerland. Acarologia. (2002) 42:3–19.

[35] Krčmar S. Hard ticks (Acari, Ixodidae) of Croatia. ZooKeys. (2012) 234:19–57. 10.3897/zookeys.234.3658 23372407 PMC3496912

[36] Brinck P Svedmyr A von Zeipel G. Migrating birds at Ottenby Sweden as carriers of ticks and possible transmitters of tick-borne encephalitis virus. Oikos. (1965) 16:88–99. 10.2307/3564868

[37] Haarløv N. Variation in the ixodid tick, *Ixodes arboricola* Schulze & Schlottke 1929. Parasitology. (1962) 52:425–39. 10.1017/S0031182000027244

[38] Petney TN Pfäffle MP Skuballa JD. An annotated checklist of the ticks (Acari: Ixodida) of Germany. Syst Appl Acarol. (2012) 17:115–170. 10.11158/saa.17.2.2

[39] Ciebiera O Jerzak L Nowak-Chmura M Bocheński M. Ticks (Acari: Ixodida) on birds (Aves) migrating through the Polish Baltic coast. Exp Appl Acarol. (2019) 77:241–51. 10.1007/s10493-019-00341-z 30771037

[40] Heylen DJA Van Oosten AR Devriendt N Elst J De Bruyn L Matthysen E. Seasonal feeding activity of the tree-hole tick, *Ixodes arboricola*. Parasitology. (2014) 141:1044–51. 10.1017/S0031182014000225 24655546

[41] Nowak-Chmura M Siuda K Wegner Z Piksa K. Species diversity of ticks (Acari: Ixodida) on migrating birds on the Baltic Sea coast of Poland. Zool Stud. (2012) 51:7.

[42] Palomar AM Portillo A Crespo A Santibáñez S Mazuelas D Oteo JA. Prevalence of “*Candidatus* Rickettsia vini” in *Ixodes arboricola* ticks in the North of Spain, 2011-2013. Parasit Vectors. (2015) 8:110. 10.1186/s13071-015-0724-6 25889739 PMC4337319

[43] Garben AFM Lina PHC Jansen J Van Bronswijk J. Teken (Ixodida) van vogels gevangen te Meyendel (Gemeente's-Gravenhage). Entomol Ber. (1978) 38:156–8.

[44] Palomar AM Santibáñez P Mazuelas D Roncero L Santibáñez S Portillo A. Role of birds in dispersal of etiologic agents of tick-borne zoonoses, Spain, 2009. Emerg Infect Dis. (2012) 18:1188–91. 10.3201/eid1807.111777 22709801 PMC3376802

[45] Van Oosten AR Heylen DJA Matthysen E. Host specificity of a bird-specialised endophilic ectoparasite, the tree-hole tick *Ixodes arboricola*. Parasitol Res. (2014) 113:4397–405. 10.1007/s00436-014-4116-1 25231076

[46] Cull B Pietzsch ME Hansford KM Gillingham EL Medlock JM. Surveillance of British ticks: An overview of species records, host associations, and new records of *Ixodes ricinus* distribution. Ticks Tick-Borne Dis. (2018) 9:605–14. 10.1016/j.ttbdis.2018.01.011 29426591

[47] Hornok S Kováts D Horváth G Kontschán J Farkas R. Checklist of the hard tick (Acari: Ixodidae) fauna of Hungary with emphasis on host-associations and the emergence of *Rhipicephalus sanguineus*. Exp Appl Acarol. (2020) 80:311–28. 10.1007/s10493-019-00461-6 32030605

[48] Obsomer V Wirtgen M Linden A Claerebout E Heyman P Heylen D. Spatial disaggregation of tick occurrence and ecology at a local scale as a preliminary step for spatial surveillance of tick-borne diseases: general framework and health implications in Belgium. Parasit Vectors. (2013) 6:190. 10.1186/1756-3305-6-190 23800283 PMC3726513

[49] Kristofìk J Masan P Sustek Z Gajdos P. Arthropods in the nests of penduline tit (*Remiz pendulinus*). Biol Bratisl. (1993) 48:493.9439114

[50] Heylen DJA Matthysen E. Contrasting detachment strategies in two congeneric ticks (Ixodidae) parasitizing the same songbird. Parasitology. (2010) 137:661–7. 10.1017/S0031182009991582 20025822

[51] Siuda K Szymański S. A case of bringing along Mediterranean ticks *Ixodes (Ixodes) festai* Rondelli, 1926 (Acari: Ixodida: Ixodidae) to Poland by migrating birds. Wiad Parazytol. (1991) 37:25–9. 1823490

[52] Nebogatkin IV. Birds as the feeders of ticks (Acari, Ixodida) in megalopolis of Kyiv. Vestnik Zoologii. (2014) 48:467–70. 10.2478/vzoo-2014-0055

[53] Pietzsch ME Mitchell R Jameson LJ Morgan C Medlock JM Collins D. Preliminary evaluation of exotic tick species and exotic pathogens imported on migratory birds into the British Isles. Vet Parasitol. (2008) 155:328–32. 10.1016/j.vetpar.2008.05.006 18585865

[54] Schilling F Böttcher M Walter G. Probleme des Zeckenbefalls bei Nestlingen des Wanderfalken (*Falco peregrinus*). J Für Ornithol. (1981) 122:359–67. 10.1007/BF01652924

[55] Sándor AD Kalmár Z Matei I Ionică AM Mărcutan I-D. Urban breeding corvids as disseminators of ticks and emerging tick-borne pathogens. Vector Borne Zoonotic Dis. (2017) 17:152–4. 10.1089/vbz.2016.2054 27922802

[56] Labbé Sandelin L Tolf C Larsson S Wilhelmsson P Salaneck E Jaenson TGT. *Candidatus* Neoehrlichia mikurensis in Ticks from Migrating Birds in Sweden. PLoS ONE. (2015) 10:e0133250. 10.1371/journal.pone.0133250 26207834 PMC4514885

[57] Dubska L Literak I Kocianova E Taragelova V Sverakova V Sychra O. Synanthropic birds influence the distribution of *Borrelia* species: analysis of *Ixodes ricinus* ticks feeding on passerine birds. Appl Environ Microbiol. (2011) 77:1115–7. 10.1128/AEM.02278-10 21148704 PMC3028744

[58] Christensen ND Skirnisson K Nielsen ÓK. The Parasite Fauna of the Gyrfalcon (*Falco rusticolus*) in Iceland. J Wildl Dis. (2015) 51:929–33. 10.7589/2015-01-022 26280881

[59] Jaenson TG Jensen J. Records of ticks (Acari, Ixodidae) from the Faroe islands. Nor J Entomol. (2007) 54:11.

[60] Santos-Silva MM Beati L Santos AS De Sousa R Núncio MS Melo P. The hard-tick fauna of mainland Portugal (Acari: Ixodidae): an update on geographical distribution and known associations with hosts and pathogens. Exp Appl Acarol. (2011) 55:85–121. 10.1007/s10493-011-9440-x 21452063

[61] Klaus C Gethmann J Hoffmann B Ziegler U Heller M Beer M. Tick infestation in birds and prevalence of pathogens in ticks collected from different places in Germany. Parasitol Res. (2016) 115:2729–40. 10.1007/s00436-016-5022-5 27048511 PMC4914531

[62] Nowak-Chmura M. *Ixodes eldaricus* Djaparidze, 1950 (Ixodidae) on migrating birds–reported first time in Poland. Vet Parasitol. (2012) 186:399–402. 10.1016/j.vetpar.2011.11.029 22142944

[63] Toma L De Liberato C Magliano A Montemaggiori A Di Luca M Mereu Piras P. Recenti segnalazioni di *Ixodes festai* in Sardegna. Boll Dell'Associazione Romana Entomol. (2014) 69:1–5.

[64] Contini C Palmas C Seu V Stancampiano L Usai F. Redescription of the male of *Ixodes festai* Rondelli, 1926 (Ixodida: Ixodidae) on specimens from Sardinia (Italy). Parasite Paris Fr. (2011) 18:235–40. 10.1051/parasite/2011183235 21894264 PMC3671470

[65] Hornok S Flaisz B Takács N Kontschán J Csörgo T Csipak Á. Bird ticks in Hungary reflect western, southern, eastern flyway connections and two genetic lineages of Ixodes frontalis and Haemaphysalis concinna. Parasit Vectors. (2016) 9:101. 10.1186/s13071-016-1365-0 26912331 PMC4765043

[66] Osacar-Jimenez JJ Estrada-Peña A Lucientes-Curdi J. Ticks (Acarina: Ixodidae) of wild birds in the Ebro Middle basin (North-east Spain). Acarologia. (1998) 39:23–31.

[67] Chastel C Guiguen C Chastel O Beaucournu JC. Pouvoir pathogène, rôle vecteur et hôtes nouveaux d'*Ixodes pari* (= *I*. frontalis)(Acari; Ixodoidea; Ixodidae). Ann Parasitol Hum Comparée. (1991) 66:27–32. 10.1051/parasite/199166127

[68] Doby JM. Contribution to the knowledge of *Ixodes (Trichotoixodes) pari* Leach (= *I*. frontalis (Panzer)](Acari: Ixodidae), a tick specific to birds. Acarologia. (1999) 39:315–25.

[69] Literak I Norte AC Núncio MS de Carvalho IL Ogrzewalska M Nováková M. Ticks on passerines from the Archipelago of the Azores as hosts of borreliae and rickettsiae. Ticks Tick-Borne Dis. (2015) 6:607–10. 10.1016/j.ttbdis.2015.05.003 26013915

[70] Heylen D Fonville M Docters van Leeuwen A Stroo A Duisterwinkel M van Wieren S. Pathogen communities of songbird-derived ticks in Europe's low countries. Parasit Vectors. (2017) 10:497. 10.1186/s13071-017-2423-y 29047399 PMC5648423

[71] Palomar AM Portillo A Santibáñez P Mazuelas D Roncero L García-Álvarez L. Detection of tick-borne *Anaplasma bovis, Anaplasma phagocytophilum* and *Anaplasma centrale* in Spain. Med Vet Entomol. (2015) 29:349–53. 10.1111/mve.12124 26111122

[72] Palomar AM Portillo A Santibáñez P Mazuelas D Roncero L Gutiérrez Ó. Presence of *Borrelia turdi* and *Borrelia valaisiana* (Spirochaetales: Spirochaetaceae) in ticks removed from birds in the North of Spain, 2009-2011. J Med Entomol. (2017) 54:243–6. 10.1093/jme/tjw158 28082654

[73] Wilhelmsson P Jaenson TGT Olsen B Waldenström J Lindgren P-E. Migratory birds as disseminators of ticks and the tick-borne pathogens *Borrelia* bacteria and tick-borne encephalitis (TBE) virus: a seasonal study at Ottenby Bird Observatory in South-eastern Sweden. Parasit Vectors. (2020) 13:607. 10.1186/s13071-020-04493-5 33272317 PMC7713317

[74] Drehmann M Chitimia-Dobler L Lindau A Frank A Mai S Fachet K. *Ixodes frontalis*: a neglected but ubiquitous tick species in Germany. Exp Appl Acarol. (2019) 78:79–91. 10.1007/s10493-019-00375-3 31093856

[75] Thompson GB Arthur DR. VI.—Records of ticks collected from birds in the British Isles.−2. Ann Mag Nat Hist. (1955) 8:57–60. 10.1080/00222935508651824

[76] Thompson GB Arthur DR. XLIV.—Records of ticks collected from birds in the British Isles.−3. Ann Mag Nat Hist. (1956) 9:385–90. 10.1080/00222935608655830

[77] Homsher PJ Sonenshine DE. Scanning electron microscopy of ticks for systematic studies. 2. Structure of Haller's organ in Ixodes brunneus and Ixodes frontalis. J Med Entomol. (1977) 14:93–7. 10.1093/jmedent/14.1.93 903942

[78] Mannelli A Nebbia P Tramuta C Grego E Tomassone L Ainardi R. *Borrelia burgdorferi* sensu lato infection in larval *Ixodes ricinus* (Acari: Ixodidae) feeding on blackbirds in northwestern Italy. J Med Entomol. (2005) 42:168–75. 10.1093/jmedent/42.2.168 15799526

[79] Lommano E Dvorák C Vallotton L Jenni L Gern L. Tick-borne pathogens in ticks collected from breeding and migratory birds in Switzerland. Ticks Tick-Borne Dis. (2014) 5:871–82. 10.1016/j.ttbdis.2014.07.001 25113989

[80] Jameson LJ Morgan PJ Medlock JM Watola G Vaux AGC. Importation of *Hyalomma marginatum*, vector of Crimean-Congo haemorrhagic fever virus, into the United Kingdom by migratory birds. Ticks Tick-Borne Dis. (2012) 3:95–9. 10.1016/j.ttbdis.2011.12.002 22300969

[81] Cafiso A Bazzocchi C De Marco L Opara MN Sassera D Plantard O. Molecular screening for *Midichloria* in hard and soft ticks reveals variable prevalence levels and bacterial loads in different tick species. Ticks Tick-Borne Dis. (2016) 7:1186–92. 10.1016/j.ttbdis.2016.07.017 27521265

[82] Marie-Angèle P Lommano E Humair P-F Douet V Rais O Schaad M. Prevalence of *Borrelia burgdorferi* sensu lato in ticks collected from migratory birds in Switzerland. Appl Environ Microbiol. (2006) 72:976–9. 10.1128/AEM.72.1.976-979.2006 16391149 PMC1352204

[83] Chastel C Monnat JY Le Lay G Beaucournu JC. Syndrome neurologique mortel chez une tourterelle turque (*Streptopelia decaocto*) et fixation de la tique (*Ixodes pari (*= *I*. frontalis). Ann Parasitol Hum Comparée. (1981) 56:349–51. 10.1051/parasite/1981563349 7332635

[84] Battisti E Urach K HodŽić A Fusani L Hufnagl P Felsberger G. Zoonotic pathogens in ticks from migratory birds, Italy. Emerg Infect Dis. (2020) 26:2986–8. 10.3201/eid2612.181686 33219656 PMC7706978

[85] Santos-Silva MM Sousa R Santos AS Melo P Encarnação V Bacellar F. Ticks parasitizing wild birds in Portugal: detection of *Rickettsia aeschlimannii, R. helvetica* and *R. massiliae*. Exp Appl Acarol. (2006) 39:331–8. 10.1007/s10493-006-9008-3 16897568

[86] Millán J Gortazar C Martín-Mateo MP Villafuerte R. Comparative survey of the ectoparasite fauna of wild and farm-reared red-legged partridges (*Alectoris rufa*), with an ecological study in wild populations. Parasitol Res. (2004) 93:79–85. 10.1007/s00436-004-1113-9 15103557

[87] Calvete C Estrada R Lucientes J Estrada A. Ectoparasite ticks and chewing lice of red-legged partridge, *Alectoris rufa*, in Spain. Med Vet Entomol. (2003) 17:33–7. 10.1046/j.1365-2915.2003.00402.x 12680922

[88] Morozov A Proca A. Ticks (Acari, Ixodidae) on Indigenous and Migratory Birds in Chishinau. Int Conf Young Res, X, Chisinau, Moldova. (2012).

[89] Saratsiotis A. Etude morphologique et observations biologiques sur *Ixodes gibbosus* Nuttall, 1916. Ann Parasitol Hum Comparée. (1970) 45:661–75. 10.1051/parasite/1970455661 5502997

[90] Estrada Pena A Lucientes Curdi J Castillo Hernandez JA Sanchez Acedo C Galmes Femenias M. *Alectoris rufa*, nuevo hospedador para *Ixodes ventalloi* e *Ixodes (Pholeoixodes) hexagonus*, en España. Rev Iber Parasitol 1941. (1984) 44:215–6.

[91] Matulaityte V Paulauskas A Bratchikov M Radzijevskaja J. New record of *Rickettsia vini* in *Ixodes lividus* ticks from Lithuania. Ticks Tick-Borne Dis. (2020) 11:101372. 10.1016/j.ttbdis.2020.101372 31983628

[92] Matulaityte V Radzijevskaja J Paulauskas A. First records of *Ixodes lividus* from sand martin (*Riparia riparia*) nests in Lithuania. J Vector Ecol. (2017) 42:264–70. 10.1111/jvec.12266 29125245

[93] Movila A Gatewood A Toderas I Duca M Papero M Uspenskaia I. Prevalence of *Borrelia burgdorferi* sensu lato in *Ixodes ricinus* and *I. lividus* ticks collected from wild birds in the Republic of Moldova. Int J Med Microbiol. (2008) 298:149–53. 10.1016/j.ijmm.2007.12.009

[94] Müller J Clupa W Seelig K-J. Zum Vorkommen von *Ixodes lividus* Koch (syn. *I. plumbeus* Leach) auf Uferschwalben, Riparia riparia (L.), im Kreis Staßfurt. Hercynia-Ökol Umw Mitteleur. (1975) 12:320–4.

[95] Nováková M Heneberg P Heylen DJA Medvecký M Muñoz-Leal S Šmajs D Literák I. Isolated populations of *Ixodes lividus* ticks in the Czech Republic and Belgium host genetically homogeneous *Rickettsia vini*. Ticks Tick-Borne Dis. (2018) 9:479–84. 10.1016/j.ttbdis.2017.12.018 29373306

[96] Ulmanen I Saikku P Vikberg P Sorjonen J. *Ixodes lividus* (Acari) in sand martin colonies in Fennoscandia. Oikos. (1977) 1977:20–6. 10.2307/3543318

[97] Graham RI Mainwaring MC Du Feu R. Detection of spotted fever group *Rickettsia* spp. from bird ticks in the U.K. Med Vet Entomol. (2010) 24:340–3. 10.1111/j.1365-2915.2010.00886.x 20546129

[98] Alves MAS. Effects of ectoparasites on the sand martin *Riparia riparia* nestlings. Ibis. (1997) 139:494–6. 10.1111/j.1474-919X.1997.tb04664.x

[99] Isabel LDC Cláudia NA. First report of *Ixodes lividus* (Koch, 1844) in sand martins *Riparia riparia* in Portugal. Syst Appl Acarol. (2020) 25:1883–8. 10.11158/saa.25.10.11

[100] Szép T Møller AP. Cost of parasitism and host immune defence in the sand martin *Riparia riparia*: a role for parent-offspring conflict? Oecologia. (1999) 119:9–15. 10.1007/s004420050755 28308163

[101] Szép T Møller AP. Exposure to ectoparasites increases within-brood variability in size and body mass in the sand martin. Oecologia. (2000) 125:201–7. 10.1007/s004420000447 24595831

[102] Nowak-Chmura M Siuda K. Ticks of Poland. Review of contemporary issues and latest research. Ann Parasitol. (2012) 58:125–55. 23444797

[103] Waldenström J Lundkvist A Falk KI Garpmo U Bergström S Lindegren G. Migrating birds and tickborne encephalitis virus. Emerg Infect Dis. (2007) 13:1215–8. 10.3201/eid1308.061416 17953095 PMC2828075

[104] Geller J Nazarova L Katargina O Leivits A Järvekülg L Golovljova I. Tick-borne pathogens in ticks feeding on migratory passerines in Western part of Estonia. Vector Borne Zoonotic Dis Larchmt N. (2013) 13:443–8. 10.1089/vbz.2012.1054 23590318 PMC3700471

[105] Comstedt P Bergström S Olsen B Garpmo U Marjavaara L Mejlon H. Migratory passerine birds as reservoirs of Lyme borreliosis in Europe. Emerg Infect Dis. (2006) 12:1087–95. 10.3201/eid1207.060127 16836825 PMC3291064

[106] Elfving K Olsen B Bergström S Waldenström J Lundkvist A Sjöstedt A. Dissemination of spotted fever rickettsia agents in Europe by migrating birds. PLoS ONE. (2010) 5:e8572. 10.1371/journal.pone.0008572 20052286 PMC2797135

[107] Baines D Becker M Hart S. Sheep tick Ixodes ricinus management on Welsh hill farms of designated conservation importance: implications for nationally declining birds. Med Vet Entomol. (2019) 33:352–9. 10.1111/mve.12368 30773654

[108] Wodecka B Rymaszewska A Skotarczak B. Host and pathogen DNA identification in blood meals of nymphal Ixodes ricinus ticks from forest parks and rural forests of Poland. Exp Appl Acarol. (2014) 62:543–55. 10.1007/s10493-013-9763-x 24352572 PMC3933768

[109] Kjelland V Stuen S Skarpaas T Slettan A. *Borrelia burgdorferi* sensu lato in *Ixodes ricinus* ticks collected from migratory birds in Southern Norway. Acta Vet Scand. (2010) 52:59. 10.1186/1751-0147-52-59 21054890 PMC2988791

[110] Arthur DR. XXXV.—Ticks collected from birds in Sweden. Ann Mag Nat Hist. (1952) 5:305–8. 10.1080/00222935208654296

[111] Hasle G Leinaas HP Røed KH Øines Ø. Transport of *Babesia venatorum*-infected *Ixodes ricinus* to Norway by northward migrating passerine birds. Acta Vet Scand. (2011) 53:41. 10.1186/1751-0147-53-41 21699719 PMC3132728

[112] Paulauskas A Rosef O Galdikaite E Radzijevskaja J. Infestation with *Ixodes ricinus* ticks on migrating passerine birds in Lithuania and Norway. Acta Biol Univ Daugavp. (2009) 9:1–6.

[113] Radzijevskaja J Rosef O Matulaityte V Paulauskas A. *Borrelia burgdorferi* sensu lato genospecies in *Ixodes ricinus* ticks feeding on passerine birds in southern Norway. Biologija. (2016) 62:124–33. 10.6001/biologija.v62i2.3338

[114] Alekseev AN Dubinina HV Semenov AV Bolshakov CV. Evidence of ehrlichiosis agents found in ticks (Acari: Ixodidae) collected from migratory birds. J Med Entomol. (2001) 38:471–4. 10.1603/0022-2585-38.4.471 11476325

[115] Humair P-F Douet V Morán Cadenas F Schouls LM Van De Pol I Gern L. Molecular identification of bloodmeal source in *Ixodes ricinus* ticks using 12S rDNA as a genetic marker. J Med Entomol. (2007) 44:869–80. 10.1093/jmedent/44.5.869 17915521

[116] Humair PF Turrian N Aeschlimann A Gern L. *Ixodes ricinus* immatures on birds in a focus of Lyme borreliosis. Folia Parasitol (Praha). (1993) 40:237–42. 8314179

[117] James MC Furness RW Bowman AS Forbes KJ Gilbert L. The importance of passerine birds as tick hosts and in the transmission of *Borrelia burgdorferi*, the agent of Lyme disease: a case study from Scotland. Ibis. (2011) 153:293–302. 10.1111/j.1474-919X.2011.01111.x

[118] Morán Cadenas F Rais O Humair P-F Douet V Moret J Gern L. Identification of host bloodmeal source and *Borrelia burgdorferi* sensu lato in field-collected *Ixodes ricinus* ticks in Chaumont (Switzerland). J Med Entomol. (2007) 44:1109–17. 10.1093/jmedent/44.6.1109 18047213

[119] Bjöersdorff A Bergström S Massung RF Haemig PD Olsen B. *Ehrlichia*-infected ticks on migrating birds. Emerg Infect Dis. (2001) 7:877–9. 10.3201/eid0705.017517 11747702 PMC2631880

[120] Berthová L Slobodník V Slobodník R Olekšák M Sekeyová Z Svitálková Z. The natural infection of birds and ticks feeding on birds with *Rickettsia* spp. and *Coxiella burnetii* in Slovakia. Exp Appl Acarol. (2016) 68:299–314. 10.1007/s10493-015-9975-3 26477038

[121] Biernat B Stańczak J Michalik J Sikora B Cieniuch S. *Rickettsia helvetica* and *R. monacensis* infections in immature Ixodes ricinus ticks derived from sylvatic passerine birds in west-central Poland. Parasitol Res. (2016) 115:3469–77. 10.1007/s00436-016-5110-6 27164834 PMC4980418

[122] Dubska L Literak I Kocianova E Taragelova V Sychra O. Differential role of passerine birds in distribution of *Borrelia* spirochetes, based on data from ticks collected from birds during the postbreeding migration period in Central Europe. Appl Environ Microbiol. (2009) 75:596–602. 10.1128/AEM.01674-08 19060160 PMC2632145

[123] Falchi A Dantas-Torres F Lorusso V Malia E Lia RP Otranto D. Autochthonous and migratory birds as a dispersion source for *Ixodes ricinus* in southern Italy. Exp Appl Acarol. (2012) 58:167–74. 10.1007/s10493-012-9571-8 22610454

[124] Franke J Moldenhauer A Hildebrandt A Dorn W. Are birds reservoir hosts for *Borrelia afzelii*? Ticks Tick-Borne Dis. (2010) 1:109–12. 10.1016/j.ttbdis.2010.03.001 21771517

[125] Gryczyńska A Welc-Faleciak R. Long-term study of the prevalence of *Borrelia burgdorferi* s.l. infection in ticks (Ixodes ricinus) feeding on blackbirds (*Turdus merula*) in NE Poland. Exp Appl Acarol. (2016) 70:381–94. 10.1007/s10493-016-0082-x 27631764 PMC5061843

[126] Hanincová K Taragelová V Koci J Schäfer SM Hails R Ullmann AJ. Association of *Borrelia garinii* and *B. valaisiana* with songbirds in Slovakia. Appl Environ Microbiol. (2003) 69:2825–30. 10.1128/AEM.69.5.2825-2830.2003 12732554 PMC154513

[127] Hildebrandt A Fritzsch J Franke J Sachse S Dorn W Straube E. Co-circulation of emerging tick-borne pathogens in Middle Germany. Vector Borne Zoonotic Dis Larchmt N. (2011) 11:533–7. 10.1089/vbz.2010.0048 20846013

[128] Hornok S Kováts D Csörgo T Meli ML Gönczi E Hadnagy Z. Birds as potential reservoirs of tick-borne pathogens: first evidence of bacteraemia with *Rickettsia helvetica*. Parasit Vectors. (2014) 7:128. 10.1186/1756-3305-7-128 24679245 PMC3976504

[129] Hubálek Z Anderson JF Halouzka J Hájek V. Borreliae in immature *Ixodes ricinus* (Acari:Ixodidae) ticks parasitizing birds in the Czech Republic. J Med Entomol. (1996) 33:766–71. 10.1093/jmedent/33.5.766 8840682

[130] Kazarina A Japina K Keišs O Salmane I Bandere D Capligina V. Detection of tick-borne encephalitis virus in I. ricinus ticks collected from autumn migratory birds in Latvia. Ticks Tick-Borne Dis. (2015) 6:178–80. 10.1016/j.ttbdis.2014.11.011 25534819

[131] Kipp S Goedecke A Dorn W Wilske B Fingerle V. Role of birds in Thuringia, Germany, in the natural cycle of *Borrelia burgdorferi* sensu lato, the Lyme disease spirochaete. Int J Med Microbiol IJMM. (2006) 296(Suppl 40):125–8. 10.1016/j.ijmm.2006.01.001 16530003

[132] Klitgaard K Højgaard J Isbrand A Madsen JJ Thorup K Bødker R. Screening for multiple tick-borne pathogens in *Ixodes ricinus* ticks from birds in Denmark during spring and autumn migration seasons. Ticks Tick-Borne Dis. (2019) 10:546–52. 10.1016/j.ttbdis.2019.01.007 30709658

[133] Marsot M Henry P-Y Vourc'h G Gasqui P Ferquel E Laignel J. Which forest bird species are the main hosts of the tick, *Ixodes ricinus*, the vector of *Borrelia burgdorferi* sensu lato, during the breeding season? Int J Parasitol. (2012) 42:781–8. 10.1016/j.ijpara.2012.05.010 22732161

[134] Matuschka FR Spielman A. Loss of Lyme disease spirochetes from *Ixodes ricinus* ticks feeding on European blackbirds. Exp Parasitol. (1992) 74:151–8. 10.1016/0014-4894(92)90042-9 1740177

[135] Michalik J Wodecka B Skoracki M Sikora B Stańczak J. Prevalence of avian-associated *Borrelia burgdorferi* sl genospecies in *Ixodes ricinus* ticks collected from blackbirds (*Turdus merula*) and song thrushes (*T. philomelos*). Int J Med Microbiol. (2008) 298:129–38. 10.1016/j.ijmm.2008.03.004

[136] Pajoro M Pistone D Varotto Boccazzi I Mereghetti V Bandi C Fabbi M. Molecular screening for bacterial pathogens in ticks (*Ixodes ricinus*) collected on migratory birds captured in northern Italy. Fol Parasitol. (2018) 65:008. 10.14411/fp.2018.008 29925679

[137] Skotarczak B Rymaszewska A Wodecka B Sawczuk M Adamska M Maciejewska A. PCR detection of granulocytic *Anaplasma* and *Babesia* in *Ixodes ricinus* ticks and birds in west-central Poland. Ann Agric Environ Med AAEM. (2006) 13:21−23. 16841867

[138] Spitalská E Literák I Sparagano OAE Golovchenko M Kocianová E. Ticks (Ixodidae) from passerine birds in the Carpathian region. Wien Klin Wochenschr. (2006) 118:759–64. 10.1007/s00508-006-0729-4 17186172

[139] Tokarevich NK Panferova YA Freylikhman OA Blinova OV Medvedev SG Mironov SV. Coxiella burnetii in ticks and wild birds. Ticks Tick-Borne Dis. (2019) 10:377–85. 10.1016/j.ttbdis.2018.11.020 30509727

[140] Aleksandrova NI Christova I Dimitrov D Marinov MP Panayotova E Trifonova I. Records of ixodid ticks on wild birds in Bulgaria. Probl Infect Parasit Dis. (2021) 49:35–9.

[141] Capligina V Salmane I Keišs O Vilks K Japina K Baumanis V. Prevalence of tick-borne pathogens in ticks collected from migratory birds in Latvia. Ticks Tick-Borne Dis. (2014) 5:75–81. 10.1016/j.ttbdis.2013.08.007 24246709

[142] Hornok S Csörgo T de la Fuente J Gyuranecz M Privigyei C Meli ML. Synanthropic birds associated with high prevalence of tick-borne rickettsiae and with the first detection of *Rickettsia aeschlimannii* in Hungary. Vector Borne Zoonotic Dis Larchmt N. (2013) 13:77–83. 10.1089/vbz.2012.1032 23289394

[143] Franke J Fritzsch J Tomaso H Straube E Dorn W Hildebrandt A. Coexistence of pathogens in host-seeking and feeding ticks within a single natural habitat in Central Germany. Appl Environ Microbiol. (2010) 76:6829–36. 10.1128/AEM.01630-10 20729315 PMC2953012

[144] Hildebrandt A Franke J Meier F Sachse S Dorn W Straube E. The potential role of migratory birds in transmission cycles of *Babesia* spp., *Anaplasma phagocytophilum*, and *Rickettsia* spp. Ticks Tick-Borne Dis. (2010) 1:105–7. 10.1016/j.ttbdis.2009.12.003 21771516

[145] Žekiene A Paulauskas A Radzijevskaja J Jusys V. Molecular investigation of tick-borne pathogens in ticks collected on migratory birds in Lithuania. Biologija. (2011) 57:159–65. 10.6001/biologija.v57i4.1927

[146] Movila A Reye AL Dubinina HV Tolstenkov OO Toderas I Hübschen JM. Detection of *Babesia* sp. EU1 and members of spotted fever group rickettsiae in ticks collected from migratory birds at Curonian Spit, North-Western Russia. Vector-Borne Zoonotic Dis. (2011) 11:89–91. 10.1089/vbz.2010.0043 20553110

[147] Toderaş I Alekseev N Movilă A Dubinina H Sapoval A. Molecular identification of *Rickettsia japonica, Rickettsia helvetica* and *Babesia* Sp. Eu1 in ticks collected from some birds species. Bul Acad Stiinte Mold Stiint Vietii. (2008) 306:91–7.

[148] Dubska L Literak I Kverek P Roubalova E Kocianova E Taragelova V. Tick-borne zoonotic pathogens in ticks feeding on the common nightingale including a novel strain of *Rickettsia* sp. Ticks Tick-Borne Dis. (2012) 3:265–8. 10.1016/j.ttbdis.2012.06.001 22906497

[149] Hajduskova E Literak I Papousek I Costa FB Novakova M Labruna MB Zdrazilova-Dubska L. “*Candidatus* Rickettsia mendelii” a novel basal group rickettsia detected in *Ixodes ricinus* ticks in the Czech Republic. Ticks Tick-Borne Dis. (2016) 7:482–6. 10.1016/j.ttbdis.2016.02.004 26873811

[150] Hoodless AN Kurtenbach K Nuttall PA Randolph SE. Effects of tick *Ixodes ricinus* infestation on pheasant *Phasianus colchicus* breeding success and survival. Wildl Biol. (2003) 9:171–8. 10.2981/wlb.2003.046

[151] Garben AF Vos H van Bronswijk JE. *Hemaphysalis punctata* Canestrini and Fanzago 1877, a tick of pastured seadunes on the island of Texel (The Netherlands). Acarologia. (1982) 23:19–25. 7090715

[152] Fain A Vangeluwe D Degreef M Wauthy G. Observations on mites inhabiting nests of *Bubo bubo* (L.)(Strigiformes, Strigidae) in Belgium. Belg J Zool. (1993) 123:3–3.

[153] Sándor AD Milchev B Takács N Kontschán J Szekeres S Hornok S. Five ixodid tick species including two morphotypes of *Rhipicephalus turanicus* on nestlings of Eurasian eagle owl (*Bubo bubo*) from south-eastern Bulgaria. Parasit Vectors. (2021) 14:334. 10.1186/s13071-021-04832-0 34174951 PMC8235848

[154] Douglas DJ Pearce-Higgins JW. Variation in ectoparasitic sheep tick *Ixodes ricinus* infestation on European Golden Plover chicks *Pluvialis apricaria* and implications for growth and survival. Bird Study. (2019) 66:92–102. 10.1080/00063657.2019.1617234

[155] Mancini F Toma L Ciervo A Di Luca M Faggioni G Lista F. Virus investigation in ticks from migratory birds in Italy. New Microbiol. (2013) 36:433–4. 24177308

[156] Walter G Massa R. Ein Beitrag zur Ektoparasitenfauna der Zugvögel in Norditalien 1. J Appl Entomol. (1987) 103:523–7. 10.1111/j.1439-0418.1987.tb01018.x

[157] Gallizzi K. Parasite-Induced Transgenerational Effects in the Great tit (Parus major). [PhD Thesis]. Bern Univ (2007).

[158] Baines D Taylor L. Can acaricide-impregnated leg bands fitted to female red grouse reduce sheep tick parasitization of chicks and increase chick survival? Med Vet Entomol. (2016) 30:360–4. 10.1111/mve.12185 27377883

[159] Fletcher K Baines D. The effects of acaricide treatment of sheep on red grouse *Lagopus lagopus scotica* tick burdens and productivity in a multi-host system. Med Vet Entomol. (2018) 32:235–43. 10.1111/mve.12282 29194726

[160] Arthur DR. LXXXV.—The larva of *Ixodes rothschildi* Nuttall & Warburton, 1911. Ann Mag Nat Hist. (1955) 8:711–2. 10.1080/00222935508655688

[161] Nuttall PA Kelly TC Carey D Moss SR Harrap KA. Mixed infections with tick-borne viruses in a seabird colony in Eire. Arch Virol. (1984) 79:35–44. 10.1007/BF01314301 6421266

[162] Dietrich M Beati L Elguero E Boulinier T McCoy KD. Body size and shape evolution in host races of the tick Ixodes uriae. Biol J Linn Soc. (2013) 108:323–34. 10.1111/j.1095-8312.2012.02021.x

[163] Gómez-Díaz E Boulinier T Sertour N Cornet M Ferquel E McCoy KD. Genetic structure of marine *Borrelia garinii* and population admixture with the terrestrial cycle of Lyme borreliosis. Environ Microbiol. (2011) 13:2453–67. 10.1111/j.1462-2920.2011.02515.x 21651685

[164] Kempf F Boulinier T De Meeûs T Arnathau C McCOY KD. Recent evolution of host-associated divergence in the seabird tick Ixodes uriae. Mol Ecol. (2009) 18:4450–62. 10.1111/j.1365-294X.2009.04356.x 19793353

[165] Duron O Cremaschi J McCoy KD. The high diversity and global distribution of the intracellular bacterium Rickettsiella in the polar seabird tick Ixodes uriae. Microb Ecol. (2016) 71:761–70. 10.1007/s00248-015-0702-8 26573831

[166] Gylfe Å Olsen B Strasevicius D Marti Ras N Weihe P Noppa L. Isolation of Lyme disease *Borrelia* from puffins (*Fratercula arctica*) and seabird ticks (*Ixodes uriae*) on the Faeroe Islands. J Clin Microbiol. (1999) 37:890–6. 10.1128/JCM.37.4.890-896.1999 10074497 PMC84640

[167] Danchin E. The incidence of the tick parasite Ixodes uriae in Kittiwake *Rissa tridactyla* colonies in relation to the age of the colony, and a mechanism of infecting new colonies. Ibis. (1992) 134:134–41. 10.1111/j.1474-919X.1992.tb08390.x

[168] Barton TR Harris MP Wanless S. Natural attachment duration of nymphs of the tick *Ixodes uriae* (Acari: Ixodidae) on kittiwake *Rissa tridactyla* nestlings. Exp Appl Acarol. (1995) 19:499–509. 10.1007/BF00052918

[169] Boulinier T Danchin E. Population trends in Kittiwake *Rissa tridactyla* colonies in relation to tick infestation. Ibis. (1996) 138:326–34. 10.1111/j.1474-919X.1996.tb04345.x

[170] Chastel C Monnat JY Le Lay G Guiguen C Quillien MC Beaucournu JC. Studies on bunyaviridae including Zaliv Terpeniya virus isolated from *Ixodes uriae* ticks (Acarina: Ixodidae) in Brittany, France. Arch Virol. (1981) 70:357–66. 10.1007/BF01320250 6173027

[171] McCoy KD Boulinier T Tirard C. Comparative host-parasite population structures: disentangling prospecting and dispersal in the black-legged kittiwake *Rissa tridactyla*. Mol Ecol. (2005) 14:2825–38. 10.1111/j.1365-294X.2005.02631.x 16029481

[172] Finney SK Elston DA. Natural attachment duration of adult female ticks *Ixodes uriae* (Acari: Ixodidae) on free-living adult black-legged kittiwakes *Rissa tridactyla*. Exp Appl Acarol. (1999) 23:765–9. 10.1023/A:1006249309647

[173] McCoy KD Boulinier T Schjørring S Michalakis Y. Local adaptation of the ectoparasite *Ixodes uriae* to its seabird host. Evol Ecol Res. (2002) 4:441–56.

[174] Kristjánsson TÖ Jónsson JE Svavarsson J. Variation in nest composition and abundances of ectoparasites between nests in colonially breeding common eiders *Somateria mollissima*. Bird Study. (2016) 63:346–52. 10.1080/00063657.2016.1182965

[175] Elsterová J Cern? J Müllerová J Šíma R Coulson SJ Lorentzen E Strøm H Grubhoffer L. Search for tick-borne pathogens in the Svalbard Archipelago and Jan Mayen. Polar Res. (2015) 34:27466. 10.3402/polar.v34.27466

[176] Nunn MA Barton TR Wanless S Hails RS Harris MP Nuttall PA. Tick-borne Great Island Virus: (II) Impact of age-related acquired immunity on transmission in a natural seabird host. Parasitology. (2006) 132:241–53. 10.1017/S0031182005008954 16197591

[177] Olsén B Jaenson TG Noppa L Bunikis J Bergström S. A Lyme borreliosis cycle in seabirds and *Ixodes uriae* ticks. Nature. (1993) 362:340–2. 10.1038/362340a0 8455718

[178] Coulson SJ Lorentzen E Strøm H Gabrielsen GW. The parasitic tick *Ixodes uriae* (Acari: Ixodidae) on seabirds from Spitsbergen, Svalbard. Polar Res. (2009) 28:399–402. 10.1111/j.1751-8369.2009.00117.x

[179] Descamps S. Winter temperature affects the prevalence of ticks in an Arctic seabird. PLoS ONE. (2013) 8:e65374. 10.1371/journal.pone.0065374 23750259 PMC3672161

[180] Duron O Jourdain E McCoy KD. Diversity and global distribution of the *Coxiella* intracellular bacterium in seabird ticks. Ticks Tick-Borne Dis. (2014) 5:557–63. 10.1016/j.ttbdis.2014.04.003 24915875

[181] Rollins RE Schaper S Kahlhofer C Frangoulidis D Strauß AFT Cardinale M. Ticks (Acari: Ixodidae) on birds migrating to the island of Ponza, Italy, and the tick-borne pathogens they carry. Ticks Tick-Borne Dis. (2021) 12:101590. 10.1016/j.ttbdis.2020.101590 33113477

[182] Ioannou I Chochlakis D Kasinis N Anayiotos P Lyssandrou A Papadopoulos B. Carriage of *Rickettsia* spp., *Coxiella burnetii* and *Anaplasma* spp. by endemic and migratory wild birds and their ectoparasites in Cyprus. Clin Microbiol Infect. (2009) 15:158–60. 10.1111/j.1469-0691.2008.02207.x 19281460

[183] Morel P-C. Présence en France d'*Ixodes festai* Rondelli, 1926. Ann Parasitol Hum Comparée. (1959) 34:549–51. 10.1051/parasite/1959344549

[184] Pascucci I Di Domenico M Capobianco Dondona G Di Gennaro A Polci A Capobianco Dondona A. Assessing the role of migratory birds in the introduction of ticks and tick-borne pathogens from African countries: An Italian experience. Ticks Tick-Borne Dis. (2019) 10:101272. 10.1016/j.ttbdis.2019.101272 31481344

[185] Krištofík J Mašán P Šustek Z. Arthropods (Pseudoscorpionidea, Acarina, Coleoptera, Siphonaptera) in nests of the bearded tit (*Panurus biarmicus*). Biologia (Bratisl). (2007) 62:749–55. 10.2478/s11756-007-0142-0

[186] Flaisz B Sulyok KM Kováts D Kontschán J Csörgo T Csipak Á. *Babesia* genotypes in Haemaphysalis concinna collected from birds in Hungary reflect phylogeographic connections with Siberia and the Far East. Ticks Tick-Borne Dis. (2017) 8:666–70. 10.1016/j.ttbdis.2017.04.013 28499722

[187] Tovornik D Cerný V. Finding of *Haemaphysalis erinacei erinacei* Pavesi, 1884 on birds in Yugoslavia. Folia Parasitol (Praha). (1974) 21:282. 4452493

[188] Akimov IA Nebogatkin IV. Distribution of Tick *Haemaphysalis punctata* (Acari, Ixodidae) in Ukraine. Vestnik Zoologii. (2012) 46:365–70. 10.2478/v10058-012-0030-0

[189] Pérez-Eid C Cabrita J. The larva and the nymph of *Hyalomma (Hyalomma) lusitanicum* Koch, 1844 (Acari: Ixodida): morphological description, habitats, hosts. Acarologia. (2004) 43:327–35.

[190] Aeschlimann A Büttiker W. Importations de Tiques en Suisse (Acarina Ixodoidea). Bull Société Entomol Suisse. (1975) 48:69–75.

[191] Hubálek Z Sedláček P Estrada-Peña A Vojtíšek J Rudolf I. First record of *Hyalomma rufipes* in the Czech Republic, with a review of relevant cases in other parts of Europe. Ticks Tick-Borne Dis. (2020) 11:101421. 10.1016/j.ttbdis.2020.101421 32360146

[192] Akimov IA Nebogatkin IV. Distribution of the Ixodid Tick *Hyalomma marginatum* (Acari, Ixodidae) in Ukraine. Vestnik Zoologii. (2011) 45:371–4. 10.2478/v10058-011-0022-5

[193] Capek M Literak I Kocianova E Sychra O Najer T Trnka A. Ticks of the *Hyalomma marginatum* complex transported by migratory birds into Central Europe. Ticks Tick-Borne Dis. (2014) 5:489–93. 10.1016/j.ttbdis.2014.03.002 24877976

[194] England ME Phipps P Medlock JM Atkinson PM Atkinson B Hewson R. *Hyalomma* ticks on northward migrating birds in southern Spain: Implications for the risk of entry of Crimean-Congo haemorrhagic fever virus to Great Britain. J Vector Ecol. (2016) 41:128–34. 10.1111/jvec.12204 27232135

[195] Vial L Stachurski F Leblond A Huber K Vourc'h G René-Martellet M. Strong evidence for the presence of the tick *Hyalomma marginatum* Koch, 1844 in southern continental France. Ticks Tick-Borne Dis. (2016) 7:1162–7. 10.1016/j.ttbdis.2016.08.002 27568169

[196] Hoffman T Lindeborg M Barboutis C Erciyas-Yavuz K Evander M Fransson T. Alkhurma Hemorrhagic Fever Virus RNA in *Hyalomma rufipes* ticks infesting migratory birds, Europe and Asia Minor. Emerg Infect Dis. (2018) 24:879–82. 10.3201/eid2405.171369 29664386 PMC5938767

[197] Kolodziejek J Marinov M Kiss BJ Alexe V Nowotny N. The complete sequence of a West Nile virus lineage 2 strain detected in a *Hyalomma marginatum marginatum* tick collected from a song thrush (*Turdus philomelos*) in eastern Romania in 2013 revealed closest genetic relationship to strain Volgograd 2007. PLoS ONE. (2014) 9:e109905. 10.1371/journal.pone.0109905 25279973 PMC4184904

[198] Lindeborg M Barboutis C Ehrenborg C Fransson T Jaenson TGT Lindgren P-E. Migratory birds, ticks, and crimean-congo hemorrhagic fever virus. Emerg Infect Dis. (2012) 18:2095–7. 10.3201/eid1812.120718 23171591 PMC3557898

[199] Chisu V Zobba R Foxi C Pisu D Masala G Alberti A. Molecular detection and *groEL* typing of *Rickettsia aeschlimannii* in Sardinian ticks. Parasitol Res. (2016) 115:3323–8. 10.1007/s00436-016-5091-5 27130322

[200] Matsumoto K Parola P Brouqui P Raoult D. *Rickettsia aeschlimannii* in *Hyalomma* ticks from Corsica. Eur J Clin Microbiol Infect Dis. (2004) 23:732–4. 10.1007/s10096-004-1190-9 15309667

[201] Socolovschi C Reynaud P Kernif T Raoult D Parola P. Rickettsiae of spotted fever group, *Borrelia valaisiana*, and *Coxiella burnetii* in ticks on passerine birds and mammals from the Camargue in the south of France. Ticks Tick-Borne Dis. (2012) 3:355–60. 10.1016/j.ttbdis.2012.10.019 23141104

[202] Mancuso E Toma L Polci A d'Alessio SG Di Luca M Orsini M. Crimean-Congo Hemorrhagic Fever Virus genome in tick from migratory bird, Italy. Emerg Infect Dis. (2019) 25:1418–20. 10.3201/eid2507.181345 31211933 PMC6590740

[203] Snow DW Perrins CM. The Birds of the Western Palearctic. Concise Edition. Oxford Univ. Press. (1998).

[204] Kurchatov VI. Biological peculiarity of the tick *Hyalomma marginatum* Koch, vector of equine piroplasmosis. Sovetsk Vet. (1939) 16:45–6.

