# Supplementary material for: Hard ticks (Acari: Ixodidae) associated with birds in Europe: Review of literature data
Source: Front Vet Sci. 2022 Aug 25;9:928756. doi: 10.3389/fvets.2022.928756 (PMC9453168; doi:10.3389/fvets.2022.928756)
Supplement: Supplementary Figure 1 — Reported occurrence of tick species according to avian orders. The number of bird species within an avian order, from which the relevant tick species was reported is encircled next to the name of avian order, along the line which connects it to the relevant tick species. Grouping of tick species according to habit preference, and of avian orders according to habitat type, nocturnal activity (inverse characters) and typical feeding level is simplified according to predominant traits of species in Europe (8, 203). [file Data_Sheet_1.PDF]

Typical habitat (horizontal difference)		Typical feeding level (vertically)		Name of avian order	
**open** (often water-associated)	**forest**	**ground level**	**higher**		
x		x		Galliformes	
(x)		x		Anseriformes	
(x)		x		Charadriiformes	
(x)		x		Gruiformes	
(x)		x		Procellariformes	
(x)		x		Ciconiiformes	
x	x	x	x	Accipitriformes	1
x	x	x	x	Strigiformes*	5
x	x	x	x	**Passeriformes**	34
	x	x		Columbiformes	1
	x		x	Caprimulgiformes*	
	x		x	Cuculiformes	
	x		x	Falconiformes	2
	x		x	Piciformes	1
	x		x	Coraciiformes	
	x		x	Bucerotiformes	

Tick species	Tick habitat preference (questing or hunting height)
Hyalomma lusitanicum	exophilic (ground level or higher)
Hyalomma marginatum	
Hyalomma rufipes	
Haemaphysalis punctata	
Haemaphysalis concinna	
Ixodes ventalloi	
Ixodes festai	
Ixodes frontalis	
Ixodes ricinus	
Ixodes arboricola	endophilic (higher)
Ixodes caledonicus	
Ixodes acuminatus	endophilic (ground level)
Ixodes hexagonus	
Ixodes canisuga	

x		x		Galliformes	4
(x)		x		Anseriformes	
(x)		x		Charadriiformes	1
(x)		x		Gruiformes	3
(x)		x		Procellariformes	
(x)		x		Ciconiiformes	
x	x	x	x	Accipitriformes	4
x	x	x	x	Strigiformes*	2
x	x	x	x	**Passeriformes**	56
	x	x		Columbiformes	2
	x		x	Caprimulgiformes*	
	x		x	Cuculiformes	
	x		x	Falconiformes	1
	x		x	Piciformes	
	x		x	Coraciiformes	1
	x		x	Bucerotiformes	

Hyalomma lusitanicum	exophilic (ground level or higher)
Hyalomma marginatum	
Hyalomma rufipes	
Haemaphysalis punctata	
Haemaphysalis concinna	
Ixodes ventalloi	
Ixodes festai	
Ixodes frontalis	
Ixodes ricinus	
Ixodes arboricola	endophilic (higher)
Ixodes caledonicus	
Ixodes acuminatus	endophilic (ground level)
Ixodes hexagonus	
Ixodes canisuga	

x		x		Galliformes	8
(x)		x		Anseriformes	3
(x)		x		Charadriiformes	7
(x)		x		Gruiformes	3
(x)		x		Procellariformes	
(x)		x		Ciconiiformes	1
x	x	x	x	Accipitriformes	6
x	x	x	x	Strigiformes*	3
x	x	x	x	**Passeriformes**	99
	x	x		Columbiformes	2
	x		x	Caprimulgiformes*	
	x		x	Cuculiformes	1
	x		x	Falconiformes	1
	x		x	Piciformes	4
	x		x	Coraciiformes	
	x		x	Bucerotiformes	1

Hyalomma lusitanicum	exophilic (ground level or higher)
Hyalomma marginatum	
Hyalomma rufipes	
Haemaphysalis punctata	
Haemaphysalis concinna	
Ixodes ventalloi	
Ixodes festai	
Ixodes frontalis	
Ixodes ricinus	
Ixodes arboricola	endophilic (higher)
Ixodes caledonicus	
Ixodes acuminatus	endophilic (ground level)
Ixodes hexagonus	
Ixodes canisuga	

x		x		Galliformes	1
(x)		x		Anseriformes	
(x)		x		Charadriiformes	1
(x)		x		Gruiformes	
(x)		x		Procellariformes	
(x)		x		Ciconiiformes	
x	x	x	x	Accipitriformes	1
x	x	x	x	Strigiformes*	
x	x	x	x	**Passeriformes**	33
	x	x		Columbiformes	
	x		x	Caprimulgiformes*	
	x		x	Cuculiformes	
	x		x	Falconiformes	
	x		x	Piciformes	
	x		x	Coraciiformes	
	x		x	Bucerotiformes	

Hyalomma lusitanicum	exophilic (ground level or higher)
Hyalomma marginatum	
Hyalomma rufipes	
Haemaphysalis punctata	
Haemaphysalis concinna	
Ixodes ventalloi	
Ixodes festai	
Ixodes frontalis	
Ixodes ricinus	
Ixodes arboricola	endophilic (higher)
Ixodes caledonicus	
Ixodes acuminatus	endophilic (ground level)
Ixodes hexagonus	
Ixodes canisuga	

x		x		Galliformes	1
(x)		x		Anseriformes	
(x)		x		Charadriiformes	
(x)		x		Gruiformes	
(x)		x		Procellariformes	
(x)		x		Ciconiiformes	
x	x	x	x	Accipitriformes	5
x	x	x	x	Strigiformes*	5
x	x	x	x	**Passeriformes**	52
	x	x		Columbiformes	1
	x		x	Caprimulgiformes*	1
	x		x	Cuculiformes	
	x		x	Falconiformes	2
	x		x	Piciformes	
	x		x	Coraciiformes	1
	x		x	Bucerotiformes	1

Hyalomma lusitanicum	exophilic (ground level or higher)
Hyalomma marginatum	
Hyalomma rufipes	
Haemaphysalis punctata	
Haemaphysalis concinna	
Ixodes ventalloi	
Ixodes festai	
Ixodes frontalis	
Ixodes ricinus	
Ixodes arboricola	endophilic (higher)
Ixodes caledonicus	
Ixodes acuminatus	endophilic (ground level)
Ixodes hexagonus	
Ixodes canisuga	
