# Supplementary material for: Hard ticks (Acari: Ixodidae) associated with birds in Europe: Review of literature data
Source: Front Vet Sci. 2022 Aug 25;9:928756. doi: 10.3389/fvets.2022.928756 (PMC9453168; doi:10.3389/fvets.2022.928756)
Supplement: Supplementary Table 1 — List of bird host species and tick-occurrence cases extracted from publications. Bird species in red are rare vagrants or captive populations, they do not occur regularly in Europe. [file Data_Sheet_2.PDF]

Table S1. List of bird host species and tick-occurrence cases extracted from publications. Bird species in red are rare vagrants or captive populations, they do not occur regularly in Europe.

				Ixodes arboricola					*Ixodes frontalis*					*Ixodes ricinus*					*Haemaphysalis concinna*					*Hyalomma marginatum*				
	Bird species	Broad habitat category*	Soil level vs. Higher**	Larva	Nymph	Female	Male	Total	Larva	Nymph	Female	Male	Total	Larva	Nymph	Female	Male	Total	Larva	Nymph	Female	Male	Total	Larva	Nymph	Female	Male	Total
	Barn owl – *Tyto alba*	A	S	1				3			1		1											1	1			2
	Boreal owl - *Aegolius funereus*				1			1																				
	Barn swallow – *Hirundo rustica*	A	H					1							1			1							1			1
	Barred warbler – *Sylvia nisoria*	U	S											1	3			5					1					
	Bearded reedling – *Panurus biarmicus*	U	H												1			1										
	Black grouse – *Tetrao tetrix*	G	S											1	2			3										
	Black kite – *Milvus migrans*	A	H															1					1		1			1
	Black redstart – *Phoenicurus ochruros*	G	S						1				2		4			4							1			1
	Black stork – *Ciconia nigra*	W	S												1			1										
	Black-headed gull – *Chroicocephalus ridibundus*	W	S								1		1															
	Bluethroat – *Luscinia svecica*	U	S											4	9			13							2			3
	Blyth's reed warbler – *Acrocephalus dumetorum*	U	S															1										
	Bohemian waxwing – *Bombycilla garrulus*	F	H										1					1										
	Booted eagle – *Hieraaetus pennatus*	A	H										1												1			1
	Brambling – *Fringilla montifringilla*	G	S											4	4			9										
	Canada goose – *Branta canadensis*	G	S											2	2			4										
	Carrion crow – *Corvus corone*	G	S										1		1			2										
	Cetti's warbler – *Cettia cetti*	U	H						1				1	1	1			3										
1	Chicken – *Gallus gallus (domestic form)*	G	S										1				1	1										1
	Coal tit – *Periparus ater*	F	H		1			4					2	4	2			10										
	Collared flycatcher – *Ficedula albicollis*	F	H		2	2	x	7							1			2						1				1
	Common blackbird – *Turdus merula*	G	S	1	3			6	15	19	10	2	48	55	64	6	1	138	1	4			7	2	4			6
	Common buzzard – *Buteo buteo*	A	H							1			2		1			6							1			1
	Common chaffinch – *Fringilla coelebs*	G	S						4	3	6		15	34	31	4		81					1	2	1	1		4
	Common chiffchaff – *Phylloscopus collybita*	U	H						4	2			6	11	19			34						1				1
	Common coot – *Fulica atra*	W	S															1										
	Common crane – *Grus grus*	G	S															1										
	Common cuckoo – *Cuculus canorus*	F	H															1										
	Common firecrest – *Regulus ignicapilla*	F	H							2			2		1			2										
	Common grasshopper warbler – *Locustella naevia*	U	H											1	2			5	1	1			2					
	Common house martin – *Delichon urbicum*	A	H													1		2										
	Common kestrel- *Falco tinnunculus*	A	H			1		1			1		3	1	1			3						1	1			3
	Common kingfisher – *Alcedo atthis*	W	H								1		1												2			2
	Common linnet – *Carduelis cannabina*	G	S										1	1	7			9										
	Common moorhen – *Gallinula chloropus*	W	S								1		1															
	Common nightingale – *Luscinia megarhynchos*	G	S							2	1		3	8	10			23	1	1			4	2	4			6
	Common pheasant – *Phasianus colchicus*	G	S										1	3	4			7					1					
	Common quail – *Coturnix coturnix*	G	S												1			1										
	Common redpoll – *Acanthis flammea*	G	S											1	1			3										
	Common redstart – *Phoenicurus phoenicurus*	F	H	2	1			4		2			2	18	21			43			1		1		6			3
	Common reed bunting – *Emberiza schoeniclus*	G	S						1				1	3	4			8	1	1			2					
	Common rosefinch – *Carpodacus erythrinus*	G	S											1	5			8										
	Common starling – *Sturnus vulgaris*	G	S	4	4	4	1	18					2	13	16			35					1					
	Common whitethroat – *Sylvia communis*	U	H							6	2		8	19	25	1		48							4			5
	Common wood pigeon – *Columba palumbus*	G	S	1				1		1			2															
	Corn bunting – *Emberiza calandra*	G	S															1					1	1				1
	Corn crake – *Crex crex*	G	S											1	1			4										
	Crested lark – *Galerida cristata*	G	S																					1	1			2
	Dunnock – *Prunella modularis*	U	S						1		3		4	23	30			56	3	1			5		1			1
	Eastern olivaceous warbler – *Iduna pallida*	U	S																						1			1
	Eastern woodchat shrike - *Lanius senator niloticus*																								1			1
	Eurasian blackcap – *Sylvia atricapilla*	F	H						5	6	3		16	30	30			66	3	2			6	1	2			3
	Eurasian blue tit – *Cyanistes caeruleus*	F	H	6	10	5	1+x	28	6	1	5		13	13	11			31					1		2			2
	Eurasian bullfinch – *Pyrrhula pyrrhula*	G	S					1			2		2	4	8			14										
	Eurasian collared dove – *Streptopelia decaocto*	G	S								4		4					1										
	Eurasian curlew – *Numenius arquata*	W	S											2	1			3										
	Eurasian eagle-owl – *Bubo bubo*	A	S											1	2			3						1	3		1	4
	Eurasian golden oriole – *Oriolus oriolus*	F	H											1	1			2		1			1					
	Eurasian hoopoe – *Upupa epops*	G	S											1	2			3										1

	Eurasian jay – *Garrulus glandarius*	F	H					1	1		1		5	8	12			24							1			1
	Eurasian magpie – *Pica pica*	G	S										1	1	2	2		10					1					
	Eurasian nuthatch – *Sitta europaea*	F	H	2	5		x	13					1	8	6	1		20					1					
	Eurasian oystercatcher – *Haematopus ostralegus*	G	S											1	1			2										
	Eurasian penduline tit – *Remiz pendulinus*	F	H	1				1																				
	Eurasian pygmy owl – *Glaucidium passerinum*	A	H	1				1																				
	Eurasian reed warbler – *Acrocephalus scirpaceus*	U	H					1	2	3	1		6	7	17			25	2	2			5	1	2	2	1	7
	Eurasian scops owl – *Otus scops*	F	H																					1	1			2
	Eurasian siskin – *Carduelis spinus*	G	S		1			1						4	5			9										
	Eurasian sparrowhawk – *Accipiter nisus*	A	H										1	2	7			10										
	Eurasian stonechat – *Saxicola torquatus*	G	S															1						1	3			4
	Eurasian tree sparrow – *Passer montanus*	G	S					3			1		2	3	4			10					1		1			1
	Eurasian treecreeper – *Certhia familiaris*	F	H	1				2					1	4	4			9										
	Eurasian woodcock – *Scolopax rusticola*	G	S												1			3					1					
	Eurasian wryneck – *Jynx torquilla*	G	S															1										
	European crested tit – *Lophophanes cristatus*	F	H											1	2			3										
	European golden plover – *Pluvialis apricaria*	G	S															1										
	European goldfinch – *Carduelis carduelis*	G	S								1		1		1			3										
	European green woodpecker – *Picus viridis*	F	H															1										
	European greenfinch – *Carduelis chloris*	G	S	1				1	3		4		8	7	12			23	1	1			3	1	3			4
	European herring gull – *Larus argentatus*	W	S												1			1										
	European honey buzzard – *Pernis apivorus*	G	S															1										
	European nightjar – *Caprimulgus europaeus*	G	S																						1			1
	European pied flycatcher – *Ficedula hypoleuca*	F	H		2			4					1	5	4			11						1				1
	European robin – *Erithacus rubecula*	G	S	2	5			8	14	13	6	1	34	50	54	2		119	2	2			6	2	6			8
	European serin – *Serinus serinus*	G	S		1	1		2						1	3			5										
	European turtle dove – *Streptopelia turtur*	G	S																					1				1
	Fieldfare -*Turdus pilaris*	G	S						1				2	6	10			21										
	Finsch's wheatear – *Oenanthe finschii*	G	S																					1	1			2
	Garden warbler – *Sylvia borin*	U	H											3	12			18										
	Goldcrest – *Regulus regulus*	F	H							1			2	5	8			15										
	Golden eagle – *Aquila chrysaetos*	A	H													1		1										
	Great reed warbler – *Acrocephalus arundinaceus*	U	H												4			5					1	1	2			3
	Great spotted woodpecker – *Dendrocopos major*	F	H					1						2	2			5										
	Great tit – *Parus major*	F	H	11	18	8	x	44	8	4	4		17	35	44	2	1	85	1	2			5	1	2			3
	Greenish warbler – *Phylloscopus trochiloides*	U	H											1	2			3										
	Grey partridge – *Perdix perdix*	G	S										1	1	2			5										
	Grey wagtail – *Motacilla cinerea*	G	S							1			1	1	2			3										
1	Harris's hawk – *Parabuteo unicinctus*	G	S								1		1															
	Hawfinch – *Coccothraustes coccothraustes*	G	S											8	17			27	1	1			5					
	Hazel grouse – *Tetrastes bonasia*	G	S															1										
	Hooded crow – *Corvus cornix*	G	S															2										
	House sparrow – *Passer domesticus*	G	S					2	1	1	4		7	2	3			8					1	1	1			2
	Iberian chiffchaff – *Phylloscopus ibericus*	U	H											1				1										
	Iberian grey shrike – *Lanius meridionalis*	F	H																						1			1
	Icterine warbler – *Hippolais icterina*	F	H											5	7			13										
	Lesser kestrel – *Falco naumanni*	A	H																						1			1
	Lesser redpoll – *Carduelis cabaret*	G	S								2		2	1	3			4										
	Lesser whitethroat – *Sylvia curruca*	U	H											5	13			18					1	1				1
	Little owl – *Athene noctua*	A	H		1	1		3																	1			2
	Long-eared owl – *Asio otus*	A	H						1	1	1		4		1			3										
	Long-tailed tit – *Aegithalos caudatus*	F	H	1				1		2	4		6	1				1										
	Mallard – *Anas platyrhynchos*	W	S															1										
	Marsh tit – *Poecile palustris*	F	H	2	1			5					1	3	6			11										
	Marsh warbler – *Acrocephalus palustris*	U	H											5	11			21	1	1			3	1	4			6
	Meadow pipit – *Anthus pratensis*	G	S											5	8		1	15										
	Melodious warbler – *Hippolais polyglotta*	F	H							1			1	1	1			2										
	Mew gull – *Larus canus*	W	S											1	2			3										
	Middle spotted woodpecker – *Dendrocoptes medius*	F	H															2										
	Mistle trush – *Turdus viscivorus*	F	H										2	4	4			11							2			2
	Northern goshawk – *Accipiter gentilis*	A	H	1				1							1			1							1			1
	Northern lapwing – *Vanellus vanellus*	G	S													1		2										
1	Oriental turtle dove – *Streptopelia orientalis*	G	S												1			1										
	Ortolan bunting – *Emberiza hortulana*	G	S																						1			1
1	Pechora pipit – *Anthus gustavi*	G	S															1										

	Pied wheatear – *Oenanthe pleschanka*	G	S															1										
	Peregrine falcon – *Falco peregrinus*	A	H	1	1	1		3																				
	Red crossbill – *Loxia curvirostra*	F	H												1			2										
	Red kite – *Milvus milvus*	A	H												1			1						1				1
	Red-backed shrike – *Lanius collurio*	G	S											1	4			9					2					
	Red-legged partridge – *Alectoris rufa*	G	S									1	4															
	Redwing – *Turdus iliacus*	G	S						2	4			9	13	22			40										
	Ring ouzel – *Turdus torquatus*	G	S								1		2		2			3										
	River warbler – *Locustella fluviatilis*	U	S												1			1		1			2					
	Rock bunting – *Emberiza cia*	G	S												1			1										
	Rook – *Corvus frugilegus*	G	S	1				1					1	1	1	1	1	5	1				1		1			1
	Rustic bunting – *Emberiza rustica*	G	S															1										
	Sand martin – *Riparia riparia*	A	H		1	1		3						1	1			2										
	Sardinian warbler – *Sylvia melanocephala*	U	H						1	1	1		3	1	1			2						1	1			2
	Savi's warbler – *Locustella luscinioides*	U	H											1	2			3	2	1			4		1			1
	Sedge warbler – *Acrocephalus schoenobaenus*	U	H								1		1	2	6			10	1	1			3		5	1		8
	Short-toed treecreeper – *Certhia brachydactyla*	F	H	1				2	2	2	1		5	3	2			5										
	Skylark – *Alauda arvensis*	G	S											1	4			6										
	Song thrush – *Turdus philomelos*	G	S	1	2			3	11	13	8		33	43	49			103	1	1			4	1	2			3
	Spanish Sparrow – *Passer hispaniolensis*	G	S																					1				1
	Spotless starling – *Sturnus unicolor*	G	S	1				2																				
	Spotted flycatcher – *Muscicapa striata*	F	H		1	1		2						3	3			6						1	1			2
	Spotted nutcracker – *Nucifraga caryocatactes*	F	H												1			2										
	Subalpine warbler – *Sylvia cantillans*	U	H							1			1															
	Tawny owl – *Strix aluco*	A	H	1				2										1							1			1
	Thrush nightingale – *Luscinia luscinia*	G	S											13	12			27						1				1
	Tree pipit – *Anthus trivialis*	G	S										1	15	16			35					2	1	4			5
1	Turkey – *Meleagris gallopavo (domestic form)*	G	S															1										
	Water pipit – *Anthus spinoletta*	G	S																					1				1
	Water rail – *Rallus aquaticus*	G	S								1		2															
	Western jackdaw – *Corvus monedula*	G	S	1	1	1		5						1	1			3							1			1
	Western olivaceous warbler – *Iduna opaca*	U	H																						1			1
	Western yellow wagtail – *Motacilla flava*	G	S						1	1			2	2	3			5						2	2			5
	Wheatear – *Oenanthe oenanthe*	G	S												5			6							1			2
	Whinchat – *Saxicola rubetra*	G	S							1			1		3			4							1			2
	White wagtail – *Motacilla alba*	G	S											1	2	1		4						1	3			4
	Willow Ptarmigan – *Lagopus lagopus*	G	S											3	3			6										
	Willow tit – *Poecile montana*	F	H		1			2					2	3	7			12										
	Willow warbler – *Phylloscopus trochilus*	F	H					1	4	1	1		6	19	23			44	1				1	2	1			3
	Winter wren – *Troglodytes troglodytes*	G	S		1			2	6	5	2		14	27	27	1		63	1				1					
	Wood warbler – *Phylloscopus sibilatrix*	F	H											4	2			7										
	Woodchat shrike – *Lanius senator*	A	S																					1	1			2
	Woodlark – *Lullula arborea*	G	S											2	3			5										
	Yellow-browed warbler – *Phylloscopus inornatus*	U	H															1										
	Yellowhammer – *Emberiza citrinella*	G	S					1						2	4			9	3	3			7					1
	TOTAL			45	64	26	1	199	96	101	87	4	343	622	844	24	5	1743	28	27	1		96	42	103	4	2	161

* Broad habitat categories

A - aerial hunter - a species, which hunts primarily through active flight or from high ventage point;

F - foliage gleaner - a species which looks for food in the arboreal region or the trunk of trees;

G - ground feeder - a species looking for and collecting the food on the soil;

U - undergrowth/thicket specialist - a species looking for food in dense undergrowth, or bush-level, close to, but not on the ground;

W - wetland specialist - primarily feeding in water or from water covered surfaces.

**Soil level vs. Higher

S - species regularly occurring on the soil/at the soil level;

H - species rarely occurring at soil level.
